# Neuropathy-associated *Fars2* deficiency affects neuronal development and potentiates neuronal apoptosis by impairing mitochondrial function

**DOI:** 10.1186/s13578-022-00838-y

**Published:** 2022-07-06

**Authors:** Xihui Chen, Fangfang Liu, Bowen Li, Yufeng Wang, Lijuan Yuan, Anan Yin, Qi Chen, Weihong Hu, Yan Yao, Mengjie Zhang, YuanMing Wu, Kun Chen

**Affiliations:** 1 Department of Biochemistry and Molecular Biology, School of Basic Medicine, Air Force Medical University, Xi’an, Shaanxi People’s Republic of China; 2 Shaanxi Provincial Key Laboratory of Clinic Genetics, Air Force Medical University, Xi’an, Shaanxi People’s Republic of China; 3 Department of Neurobiology, School of Basic Medicine, Air Force Medical University, Xi’an, Shaanxi People’s Republic of China; 4 Medical Genetics, Yan’an University, Yan’an, Shaanxi People’s Republic of China; 5 Department of General Surgery, Tangdu Hospital, Air Force Medical University, Xi’an, Shaanxi People’s Republic of China; 6 Department of Neurosurgery, Xijing Institute of Clinical Neuroscience, Department of Plastic surgery, Xijing Hospital, Air Force Medical University, Xi’an, Shaanxi People’s Republic of China; 7 Department of Anatomy, Histology and Embryology and K.K. Leung Brain Research Centre, School of Basic Medicine, Air Force Medical University, Xi’an, Shaanxi People’s Republic of China

**Keywords:** Mitochondrial phenylalanyl-tRNA synthetase, Neurite outgrowth, Neurondegeneration, Mitochondrial dysfunction, Oxidative phosphorylation complexes, Zebrafish model

## Abstract

**Background:**

Neurodegenerative diseases encompass an extensive and heterogeneous group of nervous system disorders which are characterized by progressive degeneration and death of neurons. Many lines of evidence suggest the participation of mitochondria dysfunction in these diseases. Mitochondrial phenylalanyl-tRNA synthetase, encoded by *FARS2*, catalyzes the transfer of phenylalanine to its cognate tRNA for protein synthesis. As a member of mt-aaRSs genes, *FARS2* missense homozygous mutation c.424G > T (p.D142Y) found in a Chinese consanguineous family first built the relationship between pure hereditary spastic paraplegia (HSP) and *FARS2* gene. More *FARS2* variations were subsequently found to cause heterogeneous group of neurologic disorders presenting three main phenotypic manifestations: infantile-onset epileptic mitochondrial encephalopathy, later-onset spastic paraplegia and juvenile onset refractory epilepsy. Studies showed that aminoacylation activity is frequently disrupt in cases with *FARS2* mutations, indicating a loss-of-function mechanism. However, the underlying pathogenesis of neuropathy-associated *Fars2* deficiency is still largely unknown.

**Results:**

Early gestation lethality of global *Fars2* knockout mice was observed prior to neurogenesis. The conditional *Fars2* knockout-mouse model delayed lethality to late-gestation, resulting in a thinner cortex and an enlarged ventricle which is consist with the MRI results revealing cortical atrophy and reduced cerebral white matter volume in *FARS2*-deficient patients. Delayed development of neurite outgrowth followed by neuronal apoptosis was confirmed in *Fars2*-knockdown mouse primary cultured neurons. Zebrafish, in which *fars2* was knocked down, exhibited aberrant motor neuron function including reduced locomotor capacity which well restored the spastic paraplegia phenotype of *FARS2*-deficient patients. Altered mitochondrial protein synthesis and reduced levels of oxidative phosphorylation complexes were detected in *Fars2*-deficient samples. And thus, reduced ATP, total NAD levels and mitochondrial membrane potential, together with increased ROS production, revealed mitochondrial dysfunction both in vitro and in vivo. *Dctn3* is a potential downstream molecule in responds to *Fars2* deficient in neurons, which may provide some evidence for the development of pathogenesis study and therapeutic schedule.

**Conclusions:**

The *Fars2* deficiency genetic models developed in this study cover the typical clinical manifestations in *FARS2* patients, and help clarify how neuropathy-associated *Fars2* deficiency, by damaging the mitochondrial respiratory chain and impairing mitochondrial function, affects neuronal development and potentiates neuronal cell apoptosis.

**Supplementary Information:**

The online version contains supplementary material available at 10.1186/s13578-022-00838-y.

## Background

Mitochondrial aminoacyl-tRNA synthetases (mt-aaRSs) are a family of enzymes that are encoded by the nuclear genome, translated by cytosolic ribosomes, and imported into the mitochondria where they charge specific tRNAs for protein synthesis [1]. Thirteen mitochondrial DNA (mt-DNA) encoded proteins are synthesized via the mitochondrial translation machinery. These proteins are subunits of respiratory chain complexes that play an important role in oxidative phosphorylation (OXPHOS), a crucial determinant of mitochondrial function [2]. Mutations in mt-aaRSs genes including *DARS2* [3], *EARS2* [4], *FARS2* [5], *MARS2* [6] and *RARS2* [7] can lead to central nervous system (CNS) disorders and/or muscle-related pathologies [8, 9]. Infantile-onset epileptic encephalopathy, later-onset spastic paraplegia and recently identified juvenile onset refractory epilepsy are three main phenotypes exhibited by patients harboring *FARS2* mutations [10]. To date, 39 pathogenic *FARS2* variants have been implicated across various disease types. We have previously shown that a missense homozygous mutation c.424G > T (p.D142Y) in the *FARS2* gene is the cause of pure-form hereditary spastic paraplegia (HSP) [11].

*FARS2* encodes mitochondrial phenylalanyl-tRNA synthetase (mtPheRS) that contains a class II catalytic domain and an anti-codon binding domain [12] that drive the aminoacylation reaction during protein synthesis in the mitochondria [13]. Patients with *FARS2* mutations who received enzymatic tests all showed reduced or even absent aminoacylation activity [14]. These findings suggest that mtPheRS loss-of-function variants that cause mitochondrial dysfunction may promote pathogenesis. Due to the lack of an animal model and the unobtainable of human samples, however, the relationship between *FARS2* deficiency and nervous system development and maintenance is unknown; research into *FARS2* deficiency therefore remains in its infancy. Functional studies of *FARS2* in *Saccharomyces cerevisiae*, have provided invaluable insights into studying *Fars2* function. Nevertheless, when considering phenotypic heterogeneity of human disease or translational relevance at the level of organs or tissues, it is clear that a single-cell microorganism is not an ideal model system in which to study human neurodegenerative diseases [15].

Therefore, to mimic the pathophysiology of *FARS2* deficiency, we established mutant p.D142Y and global *Fars2*-knockout-mouse models. Embryos genotyped as homozygous *Fars2*-knockout or expressing the p.D142Y mutation, stop developing during early gestation, just before the neural plate is formed. Furthermore, conditional *Fars2*-deletion in mouse embryos at embryonic day (E) 11 causes significant neural cell apoptosis and a lethal phenotype at late-gestation. Thinner cortex and enlarged ventricle were observed which is consist with the MRI results revealing cortical atrophy and reduced cerebral white matter volume in human patients. *In vitro Fars2*-knockdown mouse neurons showed delayed and disrupt neurite outgrowth. Both in vivo and in vitro experiments revealed disrupted mitochondrial protein synthesis, impaired OXPHOS biogenesis, and injured mitochondria. The further generated *fars2*-knockdown zebrafish models showed impaired motor axon growth and reduced locomotor capacity, features believed to be consistent with the manifestation of HSP patients.

To our knowledge, the present study is the first to demonstrate a relationship between *FARS2* deficiency and brain function using cell culture, and animal (mice and zebrafish) models. These models help elucidate the pathophysiology that underlies *FARS2*-related neurodegenerative diseases.

## Results

### Review of *FARS2* variations and human disease

**Fig. 1:**
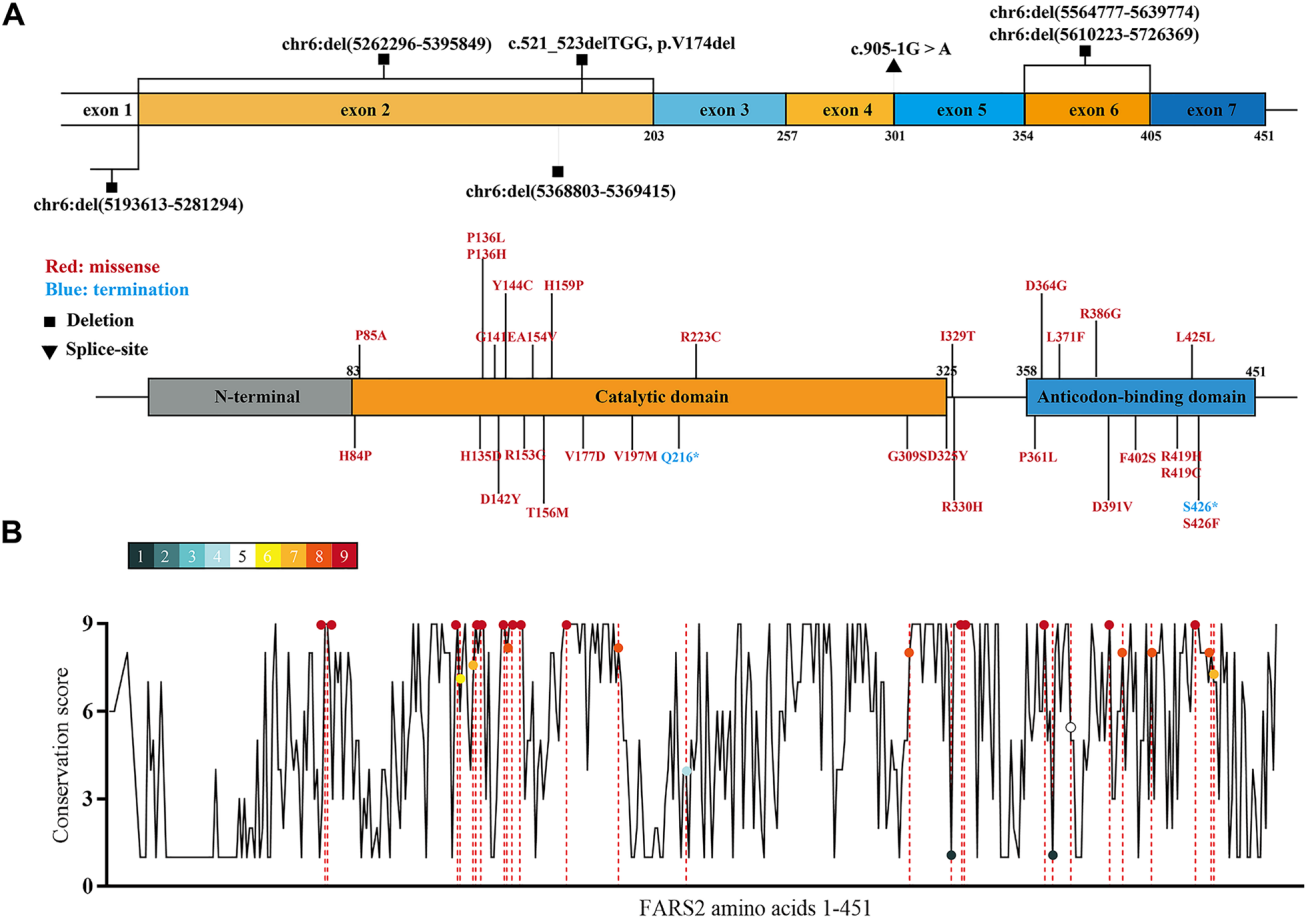
Distribution and conservation analysis of FARS2 pathogenic mutations. ** A** Distribution of 30 missense mutations, 2 nonsense mutations, 6 inframe or outframe deletions and 1 splice-site mutation on human mtPheRS. **B** A graphical illustration of mtPheRS sequence conservation (black line) based on the ConSurf conservation score. Mutation sites (dots) are indicated

The human *FARS2* gene consists of seven exons, of which exons 2–7 are responsible for coding mtPheRS. As the smallest aaRS, mtPheRS has only two structural domains: class II catalytic domain and anti-codon binding domain [16]. Here, we summarize all the variations in *FARS2* that have been reported to cause human disease to date (Table 1). These mutations are spread throughout the whole peptide of mtPheRS and include 30 missense mutations, 2 nonsense mutations, 6 inframe or outframe deletions and 1 splice-site mutation (Fig. 1A). Patients inherit these mutations in an autosomal recessive manner: compound heterozygous for two mutations or homozygous for one mutation. Conservation analysis across species showed that most of the missense mutations occur at highly conserved sites, with a ConSurf score of no less than 6. While some mutations occurred at less conserved sites, such as p.R223, p.D325, p.D364 and p.L371, can still be related to disease (Fig. 1B).

**Table 1:** Overview of *FARS2* variations related to human disease

Variations		Onset	Developmental delay	Enzyme function	OXPHOS biochemistry	References
Early-onset epileptic encephalopathy						
1	c.251 A > C (p.H84P);	10 m	+	Decreased mtPheRS abundance	↓CI and CIV in fibroblasts	[17]
	c.1256G > A (p.R419H)					
2	c.431 A > G (p.Y144C)	in infancy	+	Affected mtPheRS aminoacylation activity	NA	[5, 18, 19]
3	c.431 A > G (p.Y144C);	in infancy	NA	NA	NA	[19]
	c.530 T > A (p.V177D)					
4	c.667 C > T (p.R223C)	5 m	+	NA	NA	[20]
5	c.925G > A (p.G309S)	3/4m	+	NA	Combined OXPHO deficiency	[21]
6	c.973G > T (p.D325Y)	6 m	+	Disappared mtPheRS aminoacylation activity	↓CIV defect in myoblasts	[22]
7	c.973G > T (p.D325Y)	6 m	+	NA	NA	[18]
	chr6:del(5,193,613– 5,281,294)					
8	c.986T > C (p.I329T)	2d	+	Affected mtPheRS aminoacylation activity	NA	[5]
	c.1172 A > T (p.D391V)					
9	c.989G > A (p.R330H)	6w	+	Decreased mtPheRS abundance	↓CIV in muscle and fibroblasts	[17]
	c.1113G > T (p.L371F)					
10	c.1156 C > G (p.R386G)	3/7m	+	NA	↓CI and CIV in fibroblasts	[23]
	chr6:del(5,262,296–5,395,849)					
11	c.1255 C > T (p.R419C);	in infancy	+	NA	NA	[18]
	chr6:del(5,610,223– 5,726,369)					
12	c.1256G > A (p.R419H)	< 1 m	+	Decreased mtPheRS abundance	↓CI in muscle;↓CI and CIV in fibroblasts	[17]
	c.1269_1276del (p.S426*)					
Later-onset spastic paraplegia						
13	c.422G > A (p.G141E)	18/24m	NA	NA	NA	[24]
	chr6:del(5,564,777–5,639,774)					
14	c.424G > T (p.D142Y)	3y	-	Decreased mtPheRS aminoacylation activity	NA	[11]
15	c.461 C > T (p.A154V)	6 m	+	Decreased mtPheRS aminoacylation activity	↓CIV in muscle and fibroblasts;↓CI in fibroblasts	[25]
	c.1082 C > T (p.P361L)					
16	c.476 A > C (p.H159P)	1/2y	+	NA	NA	[19]
	c.1255 C > T (p.R419C)					
17	c.521_523del (p.V174del)	10 m	+	NA	NA	[25]
	c.1082 C > T (p.P361L**)**					
18	c.646 C > T (p.Q216*)	2.5y	-	NA	NA	[26]
	c.407 C > A (p.P136H)			Decreased mtPheRS aminoacylation activity		
19	c.1082 C > T (p.P361L)	5y	-	NA	NA	[25]
	ex. 1–2 del					
20	p.D364G	teenage	NA	Disappared mtPheRS aminoacylation activity	NA	[12]
Juvenile-onset refractory epilepsy						
21	c.253 C > G (p.P85A)	8y	+	Decrease mtPheRS stability	NA	[27]
	c.403 C > G (p.H135D)			Decrease mtPheRS aminoacylation activity		
22	c.589G > A (p.V197M)	12y	-	NA	NA	[28]
	c.1205T > C (p.F402S)					
23	c.589G > A (p.V197M)	17y	-	NA	**↑**CII and CIV in muscle	[10]
	chr6:del(5,368,803–5,369,415)					
Onset unknown						
24	c.467 C > T (p.T156M)	NA	NA	NA	NA	[29]
25	c.1275G > C (p.L425L)	NA	NA	NA	NA	[5]
	c.1277 C > T (p.S426F)					
26	c.905-1G > A	NA	NA	NA	NA	[19]
27	c.457 A > G (p.R153G)	NA	NA	NA	NA	[19]
	c.925G > A (p.G309S)					

NA, data not available

*FARS2* deficiency comprises a spectrum of disease severities. The phenotypes can be divided into three types according to the age of onset, namely infantile-onset epileptic encephalopathy, later-onset spastic paraplegia, and juvenile onset refractory epilepsy [10]. We observed that in addition to neuronal-muscle manifestations, global or neurological developmental delay is the most common phenotype of these patients. Mutants on which the aminoacylation activity measurement was carried out all showed varying degrees of disruption, the p.D325Y mutant relating to the infantile-onset epileptic encephalopathy phenotype and the p.D364G mutant relating to the later-onset spastic paraplegia even showed absolutely disappeared aminoacylation activity [12, 22]. Furthermore, in our previous study, the aminoacylation activity of HSP-associated FARS2 p.D142Y mutant was confirmed to be almost completely destroyed by purifying the recombinant human mtPheRS proteins in vitro [11], suggesting that loss of canonical FARS2 function could be a fundamental common mechanism of *FARS2*-related neuropathy in human. OXPHOS deficiency is another common phenotype of *FARS2* mutant patients. However, OXPHO complex activity differs between patients, and even between cell types of the same patient. Decreased complex I (CI) and CIV activities were mostly detected, while increased CII and CIV levels were detected in a patient with p.V197M and an exon 2 microdeletion. The variabilities in clinical presentation and biochemistry results suggest that the effect of *FARS2* deficiency can various among systems. Therefore, studying the effect of FARS2 deficiency on CNS specifically is a requisite for explaining the neuropathological mechanism of FARS2 loss of function disease.

### ***Fars2 is*expressed early during mouse embryonic development and expressed widely in multiple organs**

*Fars2* encodes mtPheRS which transfers phenylalanine to its cognate tRNA in mitochondria for protein translation, suggesting that it is required for nearly all eukaryotic cells and stages of eukaryotic development [30, 31]. To investigate the expression pattern of *Fars2* in mouse embryos throughout their development, we used time-mating to obtain embryos and/or embryonic CNS samples at seven different developmental stages. Total mRNA was extracted and *Fars2* expression levels were examined with quantitative RT-PCR (qRT-PCR). *Fars2* was expressed as early as E 7.5. The level of *Fars2* mRNA expression in the embryo and/or embryonic CNS remained stable until E 14, and then increased dramatically in the brain of E 17 embryos (Additional file 1: Fig.S1A). Protein lysates were collected from various organs from E 17 embryos and western blotting was used to determine the level of mtPheRS expression. MtPheRS is expressed and is particularly high in heart and liver, followed by kidney and the CNS (Additional file 1: Fig. S1B and C). The above results show that *Fars2* is expressed in mouse embryos at a very early stage, and is widely expressed in multiple organs in late embryonic mice.

### **Sufficient*Fars2*function is essential for embryonic neurogenesis in mice**

**Fig. 2:**
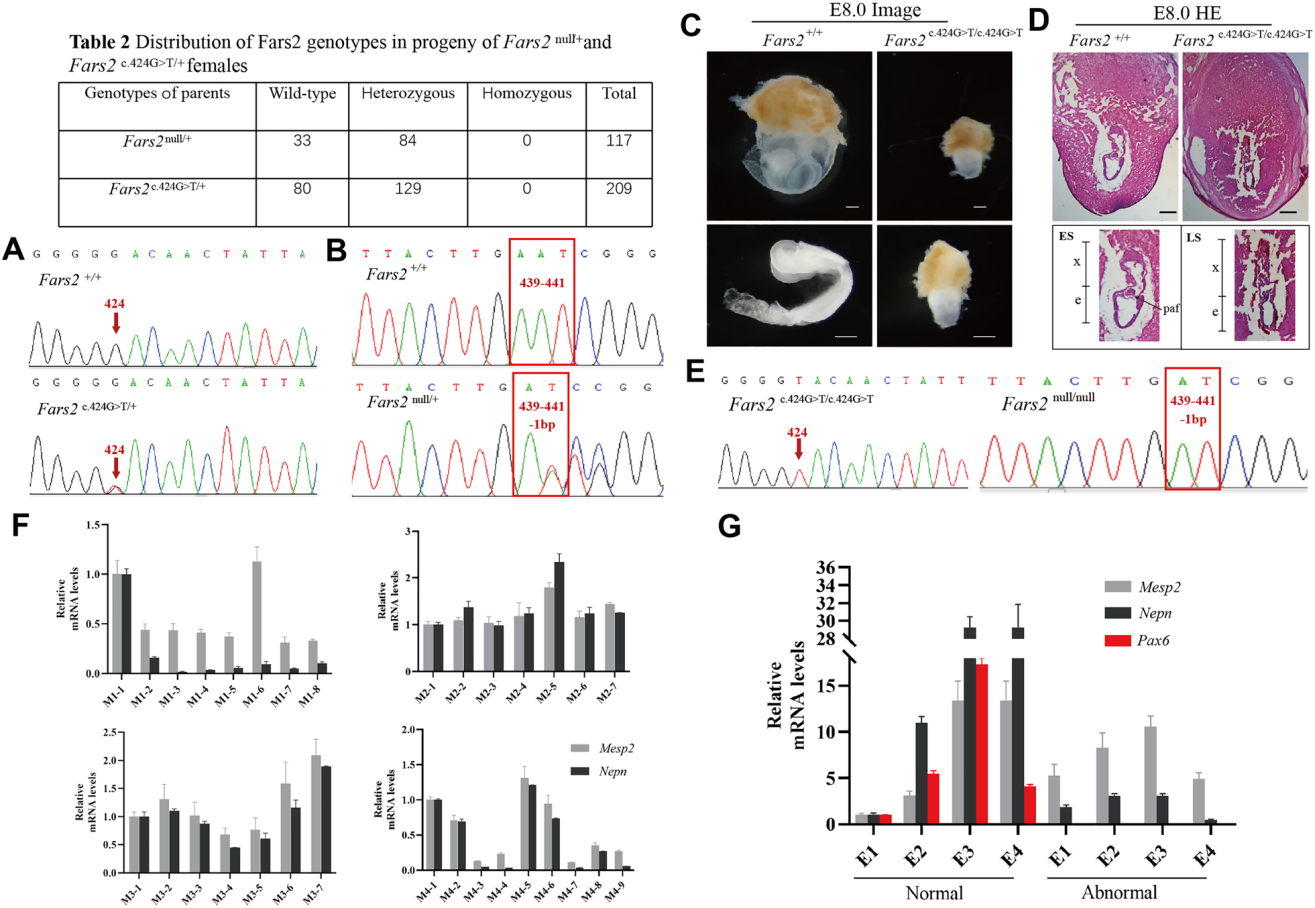
Homozygous *Fars2* knockout or mutant result in early gestation lethality in mouse. **A** Genotype of wildtype and heterozygous *Fars2* p.D142Y mutant mice, obtained by Sanger Sequencing. The red arrows indicate the 424 base site of *Fars2* cDNA sequence. **B** Genotype of wildtype and heterozygous *Fars2* knockout mice obtained by Sanger Sequencing. The red frames show the 439 to 441 base site of *Fars2* cDNA sequence. **C** Lateral view of wildtype and homozygous *Fars2* p.D142Y mutant mice embryos at E 7.5-8.0. Scale bar: 0.25 mm. **D** Hematoxylin and eosin staining of wildtype and homozygous *Fars2* p.D142Y mutant mice embryos at E 7.5-8.0. Scale bar = 0.25 mm. e, embryonic portion of egg cylinder; ES, early streak stage; LS, late streak stage; x, extraembryonic portion of the egg cylinder; paf, posterior amniotic fold. **E** Genotype of homozygous *Fars2* p.D142Y mutant and knockout mice obtained by Sanger Sequencing. The red arrows and frames respectively indicate the 424 base site and 439 to 441 base site of *Fars2* cDNA sequence. **F** qRT-PCR analysis of four E 7.0-7.5 group littermates of *Fars2* ^c.424G>T/+^ intercrossing show that all individuals express *Mesp2* and *Nepn*. **G** qRT-PCR analysis of four E 7.5-8.0 group littermates of *Fars2* ^c.424G>T/+^ intercrossing reveal that normal-shaped embryos express *Mesp2*, *Nepn*, and *Pax6*, while abnormal-shaped embryos only express *Mesp2* and *Nepn*

Our previous work confirmed that the p.D142Y mutation disrupts the aminoacylation activity of mtPheRS, leaving only a trace of enzymatic activity. To learn more about the HSP mechanism caused by this missense mutation and the physiological roles of *Fars2*, we used the CRISPR/Cas 9 system to create heterozygous global mutant and global knockout (KO) *Fars2* mice (Additional file 1: Fig. S2A). The distribution of *Fars2* genotypes in the progeny of *Fars2* ^null/+^ and *Fars2* ^c.424G>T/+^ intercrossing was counted. Genotyping confirmed that the ratio of heterozygote to wildtype was 2:1 in both mutant and KO strains, while no homozygous mutant or KO mice were born (Table 2; Fig. 2A and B). Atrophic embryos were discovered in the uterus of pregnant mice at E 14.5 subsequently, indicating embryonic lethality (Additional file 1: Fig. S2B). We used time-mating to determine the exact developmental stage of this lethal effect. During E 7.0-7.5, no obvious differences in morphology were observed between littermates, whereas abnormal appearances were observed among E 7.5-8.0 embryos derived from *Fars2* ^null/+^ and *Fars2* ^c.424G>T/+^ intercrossing (Fig. 2C, Additional file 1: Fig. S2C). Hematoxylin and eosin staining of E 7.5-8.0 embryos revealed that embryos with normal appearances had developed posterior amniotic fold structures, characteristic of embryos at the late streak stage; embryo with abnormal appearances, however, had stopped at the early streak stage and the extraembryonic portion of the egg cylinder had begun to disintegrate (Fig. 2D). Genotyping the normal and abnormal embryos confirmed them to be wildtype and homozygous *Fars2*-deficient strains (Fig. 2E). We collected four groups of littermates extracted from *Fars2* ^c.424G>T/+^ intercrossing at E 7.0-7.5 and eight litters (four normal, and four with abnormal morphology) extracted at E 7.5-8.0 to confirm the stage at which *Fars2*-deficient embryos stop developing. Total mRNA was extracted from the embryos without any maternal components. *Nepn* [32] and *Mesp2* [33] were chosen as markers of the definitive endoderm and mesoderm, and were reported to express at E 7.5 and E 7.0 respectively. In addition, *Pax6* starts to express in the neural stem/progenitor cells of ectoderm at E 8.0, the earliest stage of CNS development, and marks embryonic neurogenesis [34]. As a result, at both E 7.0-7.5 and E 7.5-8.0, all of the littermates express *Mesp2* and *Nepn*. *Pax6* expression was found in embryos with normal morphology at E 7.5-8.0, but not in embryos with a smaller appearance. These findings suggest that embryos with a homozygous *Fars2* KO or mutant genotype can develop definitive endodermal and mesodermal layers, but not the ectoderm (Fig. 2F and G). The above results indicate that *Fars2* function is required for embryonic neurogenesis, and the p.D142Y is a loss of function mutant which produces a KO-like phenotype in mice.

**Table 2:** Distribution of *Fars2* genotypes in progeny of *Fars2* ^null/+^ and *Fars2* ^c.424G>T/+^ females

Genotypes of parents	Wild-type	Heterozygous	Homozygous	Total
*Fars2* ^null/+^	33	84	0	117
*Fars2* ^c.424G>T/+^	80	129	0	209

### ***Fars2*deficiency in the CNS results in a thinner cortex and an enlarged ventricle due to progressive cell apoptosis**

**Fig. 3:**
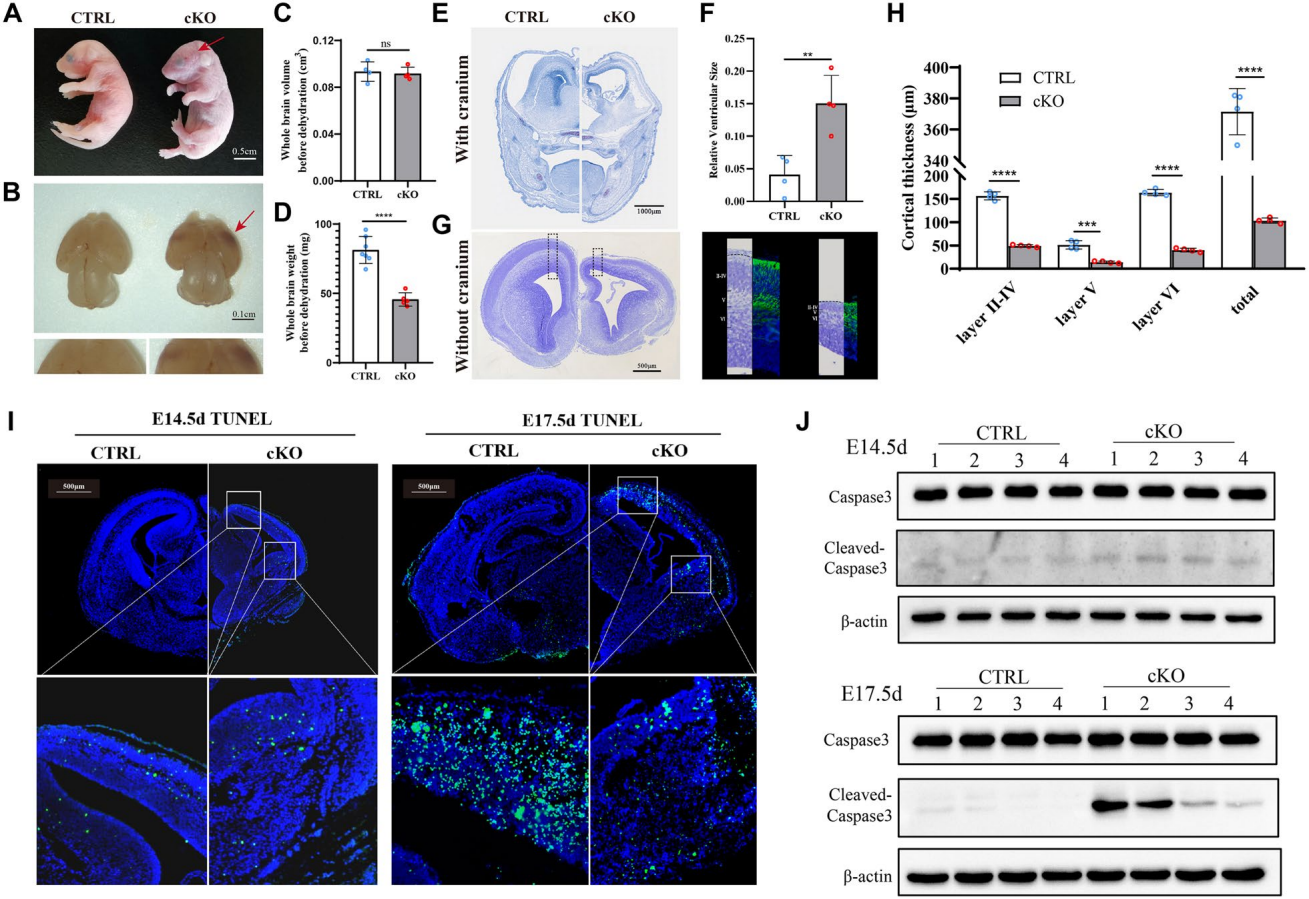
Conditional deletion of *Fars2* in mice CNS results in neurodegenerative phenotypes. **A** The appearance of control (Nes-Cre^−^; *Fars2* ^fl/fl^) and cKO (Nes-Cre^+^; *Fars2* ^fl/fl^) embryos at P 0.5. The purple appearance of the stillbirth cKO embryo is indicated by the arrow. Scale bars: 0.5 cm. **B** The appearance of the whole brain from control and cKO embryos at E 17.5. The arrow shows red lesions on cerebral cortex of a cKO embryo. Scale bars: 0.1 cm. **C** The whole brain volume of cKO embryos before dehydration was comparable with control embryos (mean ± SD; two-tailed unpaired t-Test, ns: non-significant; *n* = 4). **D** The brain weight of cKO embryos before dehydration decreased compared with control embryos (mean ± SD; two-tailed unpaired t-Test, ****P < 0.0001; *n* = 4). **E**– **F** Nissl staining of coronal sections with cranium of control and cKO mice embryo at E 17.5. Scale bars: 1000 μm. Quantification of ventricle volume showed enlarged volume in cKO embryos compared with control embryos (mean ± SD; two-tailed unpaired t-Test, **P < 0.01; *n* = 4). **G**– **H** Nissl and immunofluorescence staining of coronal sections without cranium of control and cKO mice embryo at E 15.5. Scale bars: 500 μm. The neurons were stained with TUJ1 to help distinguish cortical layers. The layer thickness of cortical areas in control and cKO embryos was quantified (mean ± SD; two-tailed unpaired t-Test, ***P < 0.001; ****P < 0.0001; *n* = 4). **I**TUNEL staining of brain sections in control and cKO mice at the indicated embryonic ages. Scale bars: 500 μm. The white frames indicate the dorsal and ventral cortex region with positive signals. **J** Western blotting for Caspase 3 and Cleaved-caspase 3 in control and cKO mice at the indicated embryonic ages

To determine if *Fars2* deletion affects brain development, we used CRISPR/Cas9 to create conditional-knockout *Fars2* mice (*Fars2* ^fl/fl^). By mating *Fars2* ^fl/fl^ mice with Nestin-Cre mice, which specifically express Cre recombinase in nervous tissue by E 11.0. Thus, neuron-specific *Fars2* knockout (cKO) mouse with the genotype of Nes-Cre^+^; *Fars2* ^fl/fl^ were generated (Additional file 1: Fig. S3A and B). Western blotting results of FARS2 in brain and liver confirmed the knockout efficiency and tissue specificity of Cre (Additional file 1: Fig. S3C). We discovered that cKO pups were born with a slight purple color and died shortly after birth (Fig. 3A). Gross examination of their cerebral cortex revealed red lesions. The total brain volume did not significantly differ between the cKO and control groups, but the weight of the brain was significantly reduced in the cKO mice (Fig. 3B-D). Frozen brain slices were cut in coronal sections to evaluate structural changes of *Fars2*-deficient embryos. At E 17.5, Nissl staining of the cortex, with and without the cranium, revealed an enlarged ventricle and reduced cortical thickness (Fig. 3E and F). Nissl staining and immunofluorescence staining for TUJ1 at E15.5 revealed that the thickness of all formed layers in the cortex were affected (Fig. 3G and H).

The TUNEL assay was then used to detect cells undergoing apoptosis. Several positive dots were scattered thinly in the dorsal cortex and ventral cortex of E 14.5 cKO mice; at E 17.5, however, the positive dots were distributed throughout these two regions (Fig. 3I). We then collected protein lysates from E 14.5 and E 17.5 embryonic cerebral cortex, and used Western blotting to determine Caspase3 and Cleaved-caspase 3 levels. Cleaved-caspase fragments of cKO were accumulated gradually (Fig. 3 J). These data suggest that the high rate of cellular apoptosis in *Fars2*-deficient mice may produce a thinner cortex, and that this process is a progressive one.

### ***Fars2*deficiency affects neurite outgrowth and causes neuron apoptosis**

**Fig. 4:**
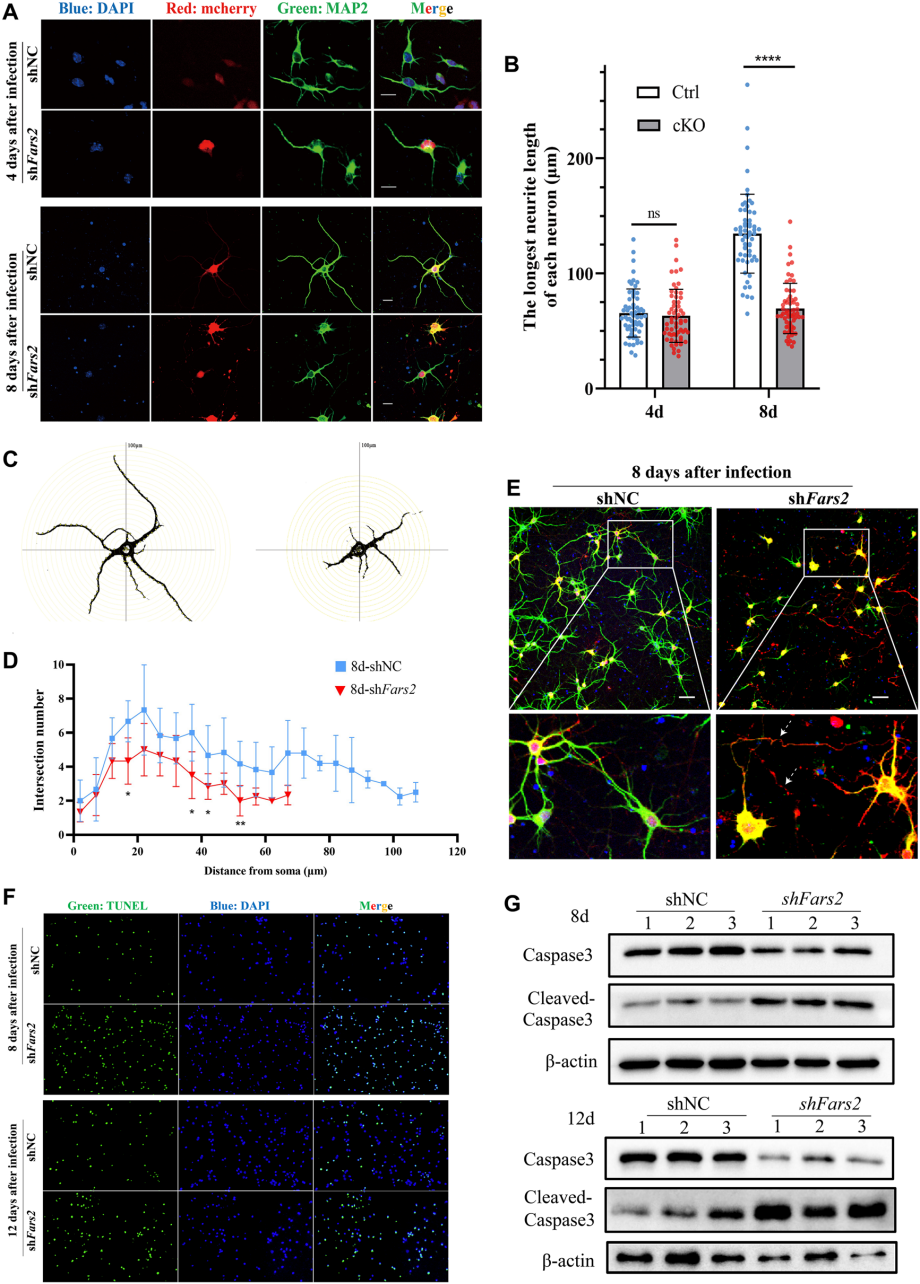
*Fars2* depletion affects neurite outgrowth in primary cultured neurons and zebrafish motor neurons. **A** Immunofluorescence staining of primary cultured neurons at the indicated day of culture. Neurons were infected with lentivirus carrying mcherry (red), the neurites were labeled with MAP2 (green) and the nucleus with DAPI (blue). Scale bars: 20 μm. **B** Quantification of the longest neurite length at the indicated days (mean ± SD; two-tailed unpaired t-Test, ns: non-significant; ****P < 0.0001; *n* = 60). **C**–**D** Sholl analysis of control and *Fars2* knocked-down neurons at the 8th day of culture (mean ± SD; two-tailed unpaired t-Test, *P < 0.5; **P < 0.01; *n* = 6). **E** Immunofluorescence staining of neurons at 8 days post-infection. Neurons were infected with lentivirus carrying mcherry (red), the neurites were labeled with MAP2 (green) and the nucleus with DAPI (blue). Arrowheads point to the fracture of axons. Scale bars: 50 μm. **F** TUNEL staining of control and *Fars2* knocked-down neurons at the indicated days of culture. **G** Western blotting for Caspase 3 and Cleaved-caspase 3 in control and *Fars2* knocked-down neurons at the indicated days in culture

To investigate the role of *Fars2* in long-term neuron development and maturation, we infected primary cultured neurons with mcherry-sh*Fars2* lentivirus and obtained neurons in which *Fars2* expression was efficiently knocked down (Additional file 1: Fig. S4A and B). Immunocytochemistry for MAP2 facilitated the tracking of neurite morphogenesis. The length of the longest primary neurite of neurons with *Fars2* knocked-down, was relatively normal following four days of culture after infection; but with a slighter figure and fewer secondary branches than in the control group. While on the eighth day, the mean length of the longest primary neurite was significantly shorter than that of control neurons, but similar to control neurons on the fourth day, indicating a development delay (Fig. 4A and B). Sholl’s analysis of concentric circles revealed that neurite arborization was significantly lower in neurons with *Fars2* knocked-down than in controls (Fig. 4C and D). Images taken with a low-power lens on the eighth day showed that connections between *Fars2*-deficient neurons were severely disrupted due to axon degeneration (Fig. 4E). In addition to morphological changes, we discovered that after long-term in vitro cultivation, very few neurons in the *Fars2-*knockdown group survived. TUNEL assays and Western blotting were used to detect apoptosis at 8 and 12 days after infection, respectively. An increased number of cell death was detected in *Fars2* knock-down group compared to the control group (Fig. 4F). And the level of Cleaved-caspase 3/Caspase 3 was significantly higher in *Fars2* knock-down neurons (Fig. 4G).

The above findings suggest that the outgrowth of neurites was severely affected by *Fars*2 deficiency, and progressive neuron apoptosis was thus triggered. As a result of delayed neurite development and devastating early apoptosis, the structural connection between *Fars2-*deficient neurons was disrupt.

### **Mitochondrial protein biosynthesis is disrupted in*Fars2*-deficient organisms**

**Fig. 5:**
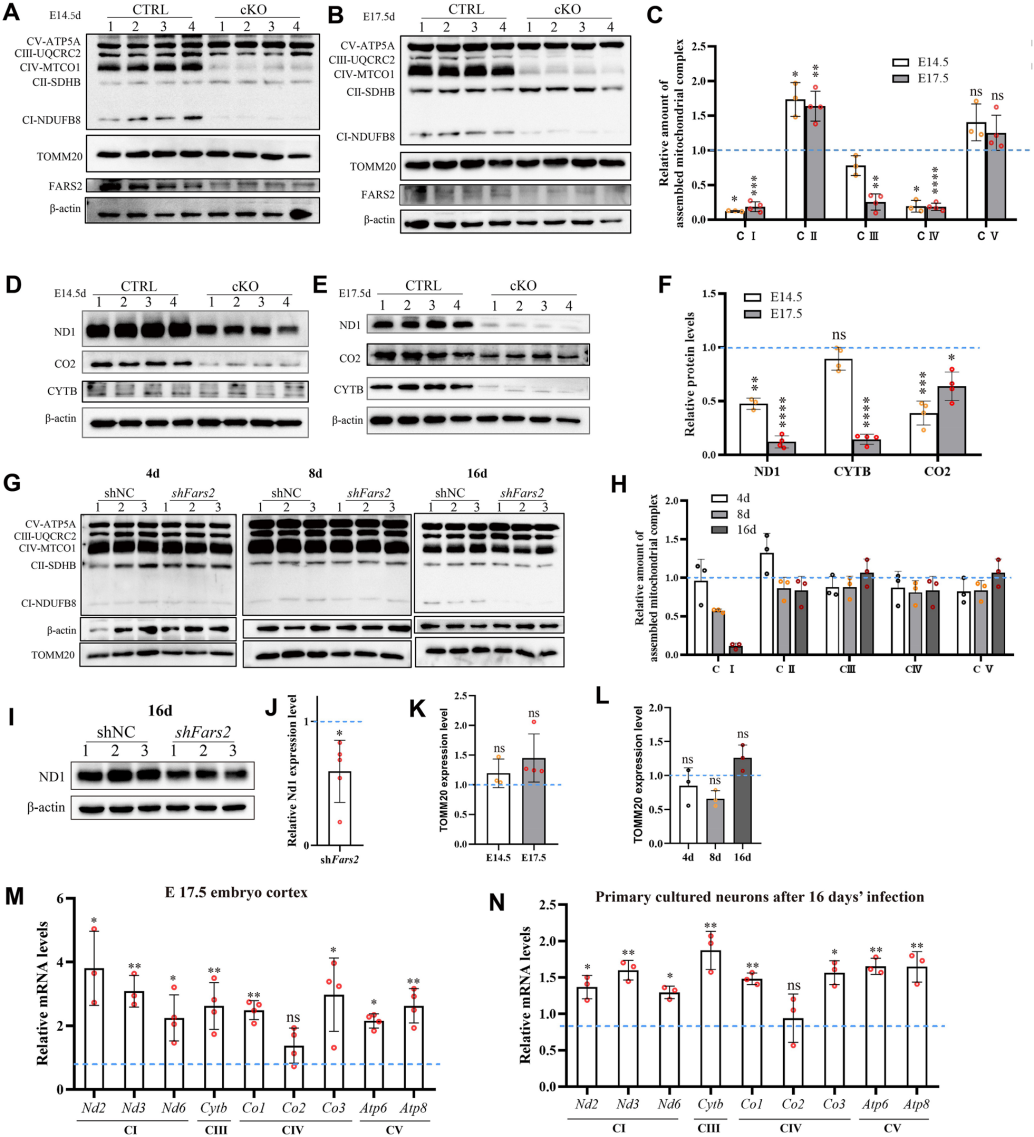
Translation and assembly of OXPHOS subunits are disrupted in *Fars2*-deficient samples. **A**–**C** Western blotting for OXPHOS complexes and the mitochondrial marker TOMM20 in control and cKO mice embryo cortex at indicated embryonic ages (mean ± SD; two-tailed unpaired t-Test, ns: non-significant; *P < 0.05; **P < 0.01; ***P < 0.001; ****P < 0.0001; *n* = 4). **D**–**F** Western blotting for ND1, CO2 and CYTB in control and cKO mice embryo cortex at the indicated embryonic ages (mean ± SD; two-tailed unpaired t-Test, ns: non-significant; *P < 0.05; **P < 0.01; ***P < 0.001; ****P < 0.0001; *n* = 4). **G**–**H** Western blotting for OXPHOS complexes and the mitochondrial marker TOMM20 in control and *Fars2* knocked-down neurons at the indicated days of culture (mean ± SD; two-tailed unpaired t-Test, ns: non-significant; *P < 0.05; *n* = 3). **I**–**J** Western blotting for ND1 in control and *Fars2* knocked-down neurons at 16 days of infection (mean ± SD; two-tailed unpaired t-Test, *P < 0.05; *n* = 3). **K**–**L** Quantification of TOMM20 in **A**, **B** and **G** (mean ± SD; two-tailed unpaired t-Test, ns: non-significant; *n* = 3–4). **M** RT-PCR analysis of OXPHOS subunits transcription levels in control and cKO mice embryo cortex at E 17.5 (mean ± SD; Two-tailed unpaired t-Test, ns: non-significant; *P < 0.05; **P < 0.01; *n* = 3–4). **N** qRT-PCR analysis of OXPHOS subunits transcription levels in control and *Fars2* knocked-down neurons at 16 days after infection (mean ± SD; Two-tailed unpaired t-Test, ns: non-significant; *P < 0.05; **P < 0.01; *n* = 3)

The mitochondrial translation machinery that mtPheRS participate in includes the synthesis of thirteen mitochondrial respiratory chain complex subunits. The proportions of phenylalanine among the five mitochondrial complexes from humans, rats, mice, and zebrafish, are shown in Additional file 1: Table S5. With the exception of CII, which lacks mitochondrial encoded subunits, phenylalanine is present in all the other four complexes. To better understand the effect of *Fars2* deficiency on protein translation, we looked at the total levels of five fully assembled respiratory chain complexes in cortex and primary cultured neurons. In E 14.5 embryos from cKO mice, Western blotting revealed significantly reduced levels of CI and CIV, as well as slightly reduced levels of CIII. At E 17.5, these three complexes were further reduced. While CII and CV levels were slightly higher in *Fars2*-deficient embryos (Fig. 5A-C). To determine whether the reduced levels were due to reduced translation of the relevant subunits, we examined the levels of the mitochondrial proteins ND1, CO2, and CYTB, which belong to the three affected complexes. Western blotting data showed consistent variation of ND1, CO2 and CYTB levels with their corresponding complexes, indicating that disruption in subunits translation is responsible for deficient mitochondrial respiratory chain complexes (Fig. 5D-F). Changes within cultured neurons after *Fars2* knockdown, however, followed different patterns. CI was almost disappeared in *Fars2* knockdown neurons after 16 days in culture, whereas CII and CV were slightly increased following 4 and 16 days of infection respectively (Fig. 5G and H); and the ND1 level showed significantly reduction in *Fars2* knockdown neurons at 16th day but can still be synthesized, which indicate that *fars2* knocking down affect both ND1 translation process and the assembly of mitochondrial CI in neurons (Fig. 5I and J). We also measured the number of mitochondria by calculating the amount of TOMM20 by Western blotting (Fig. 5A, B and G). The *Fars2*-deficient mouse cortical and cultured neurons both showed unchanged mitochondrial mass which indicated that the down-regulated complex levels cannot be attributed to the number change of mitochondria (Fig. 5K and L). Furthermore, in *Fars2*-deficient tissue and cells, transcriptional levels of mitochondrial encoded respiratory chain complex subunits were generally upregulated (Fig. 5M and N), which further demonstrates that the decreased complex levels in *Fars2*-deficient samples occurs at the post-transcriptional level.

### ***Fars2*deficiency leads to impaired OXPHOS biogenesis and damaged mitochondria**

**Fig. 6:**
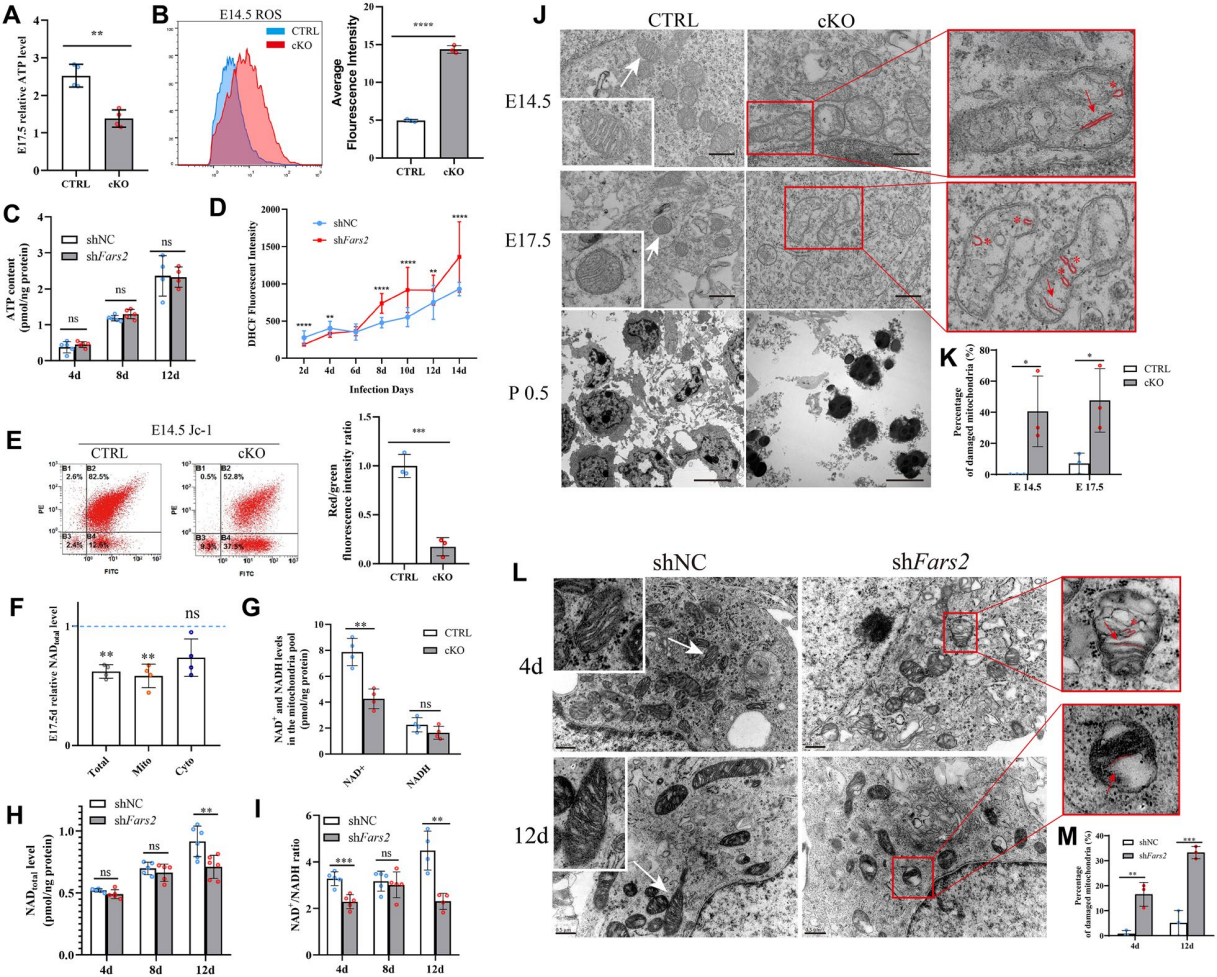
Effects of *Fars2* deficiency on mitochondria. **A**–**B** ATP and ROS levels in the cortex of the cKO mouse embryo compared with control at E 14.5 (mean ± SD; Two-tailed unpaired t-Test, **P < 0.01; ****P < 0.0001; *n* = 6 for ATP; *n* = 3 for ROS). **C**–**D** TP and ROS levels in control and *Fars2* knocked-down neurons at the indicated days of culture (mean ± SD; two-tailed unpaired t-Test, ns: non-significant; **P < 0.01; ****P < 0.0001; *n* = 4–5 for ATP; *n* = 27 for ROS). **E** Relative mitochondrial membrane potential in the cortex of the cKO mouse embryo compared with control at E 14.5 (mean ± SD; two-tailed unpaired t-Test, ***P < 0.001; *n* = 3). **F** Relative NAD_total_ level in the cortex of the cKO mouse embryo compared with control at E 17.5 (mean ± SD; two-tailed unpaired t-Test, ns: non-significant; **P < 0.01; *n* = 4). **G** NAD^+^ and NADH levels of mitochondria pool in control and cKO mice embryo cortex at E 17.5 (mean ± SD; two-tailed unpaired t-Test, ns: non-significant; **P < 0.01; *n* = 4). **H**–**I**NAD_total_ levels and NAD^+^/NADH ratio in control and *Fars2* knocked-down neurons at the indicated days of culture (mean ± SD; two-tailed unpaired t-Test, **P < 0.01; ***P < 0.001; ****P < 0.0001; *n* = 5–6). **J** Transmission electron micrograph of mitochondria in cortex from control and cKO mice embryos at the indicated embryonic ages. Scale bars: 0.5 μm for E 14.5 and E 17.5 samples and 5 μm for P 0.5 samples. **K** Percentage of mitochondria with abnormal cristae in CTRL and cKO cortex at indicated embryonic ages (mean ± SD; two-tailed unpaired t-Test, *P < 0.05; n = 3). **L** Transmission electron micrograph of mitochondria in control and *Fars2* knocked-down neurons at the indicated days of culture. Linearized mitochondrial cristae and the geometrical shapes are indicated by red lines and asterisks. Scale bars: 0.5 μm. **M** Percentage of mitochondria with abnormal cristae in control and *Fars2* knocked-down neurons at the indicated days of culture (mean ± SD; two-tailed unpaired t-Test, **P < 0.01; ***P < 0.001; n = 3)

The mitochondrial electron transfer chains are responsible for the production of almost 80% of cellular energy in the form of adenosine triphosphate (ATP), and also help regulate reactive oxygen species (ROS) [35]. We assessed the effects of reduced mitochondrial respiratory chain complexes on ATP and ROS in E 17.5 cKO cortex embryo, primary cultured neurons, and the PC12 cell line. Our findings show that *Fars2*-deficient tissue and cell lines have lower ATP concentrations and higher ROS levels, implying that mitochondrial electron transfer is dysfunctional (Fig. 6A and B; Additional file 1: Fig. S5A and B). ATP concentration in cultured neurons, however, did not change over the course of 12 days (Fig. 6C). While ROS levels were slightly lower than in controls during the first four days of *Fars2* knockdown, they began to rise steadily after the eighth day (Fig. 6D).

Increased ROS production often accompanies mitochondrial dysfunction, and reduction of the mitochondrial membrane potential (Δψm) is an associated hallmark [36, 37]. We monitored Δψm in the *Fars2*-deficient cortex and PC12 cells with JC-1 staining. Increased green fluorescence and decreased red fluorescence were detected, suggesting that mitochondria depolarization reduced Δψm (Fig. 6E; Additional file 1: Fig. S5C).

Nicotinamide adenine dinucleotide (NAD) participates in a variety of biological processes, including mitochondrial functions, oxidative stress generation, cell apoptosis, and axonal degeneration [38]. And the NAD^+^/NADH dinucleotide pair drives a wide range of reduction–oxidation reactions in cells. According to our results, the total pool of NAD level was significantly lower in the *Fars2*-deficient cortex and in cultured neurons, and the reduction is largely due to the low NAD in the mitochondria pool, but not the cytosolic pool (Fig. 6F and H). And the reduction of total NAD is largely due to the reduced NAD^+^ level in the mitochondria pool in *Fars2*-deficient mouse cortex (Fig. 6G). Interestingly, the NAD^+^/NADH ratio of cultured neurons undergo *Fars2* knockdown decreased at the 4th and 8th day of culture but seemed to be slightly rescued between the two time points (Fig. 6I).

We further used a Seahorse XF24 extracellular flux analyzer to measure mitochondrial aerobic respiration. Compared with the control group, maximal respiration and ATP production were both reduced (Additional file 1: Fig. S5D and E). This suggests that the current and potential functions of mitochondria in *Fars2*-deficient cells were disrupted. To further validate the effect on mitochondria, mitochondrial ultrastructure were directly observed using transmission electron microscopy in mouse cortex, cultured neurons and PC12 cells. In *Fars2*-deficient organisms, a generalized phenomenon was observed that mitochondrial cristae was linearized and various geometrical shapes were formed, and the phenotype deteriorated over time(Fig. 6J–M; Additional file 1: Fig. S5F and G). Images of a whole cell from 0.5 d postnatal mouse cortex even revealed a disintegration fate of *Fars2* deficiency (Fig. 6J). These morphological changes are believed to be associated with alterations in protein composition within mitochondrial protein complexes that influence inner mitochondrial membrane folding and curvature [39]. What’s more, the fate of *Fars2-*knockdown PC12 cell was measured by Annexin V/ PI staining, the results showed the number of early and late apoptosis cells were both increased in comparison to the control cells (Additional file 1: Fig. S5H).

The above findings, together, indicate that mitochondrial function is severely impaired in *Fars2*-deficient cells and animals under the condition of impaired OXPHO complexes, and that the accumulation of damaged mitochondria may eventually lead to cells apoptosis.

### **Aberrant*fars2*function causes reduced locomotor capacity in zebrafish**

**Fig. 7:**
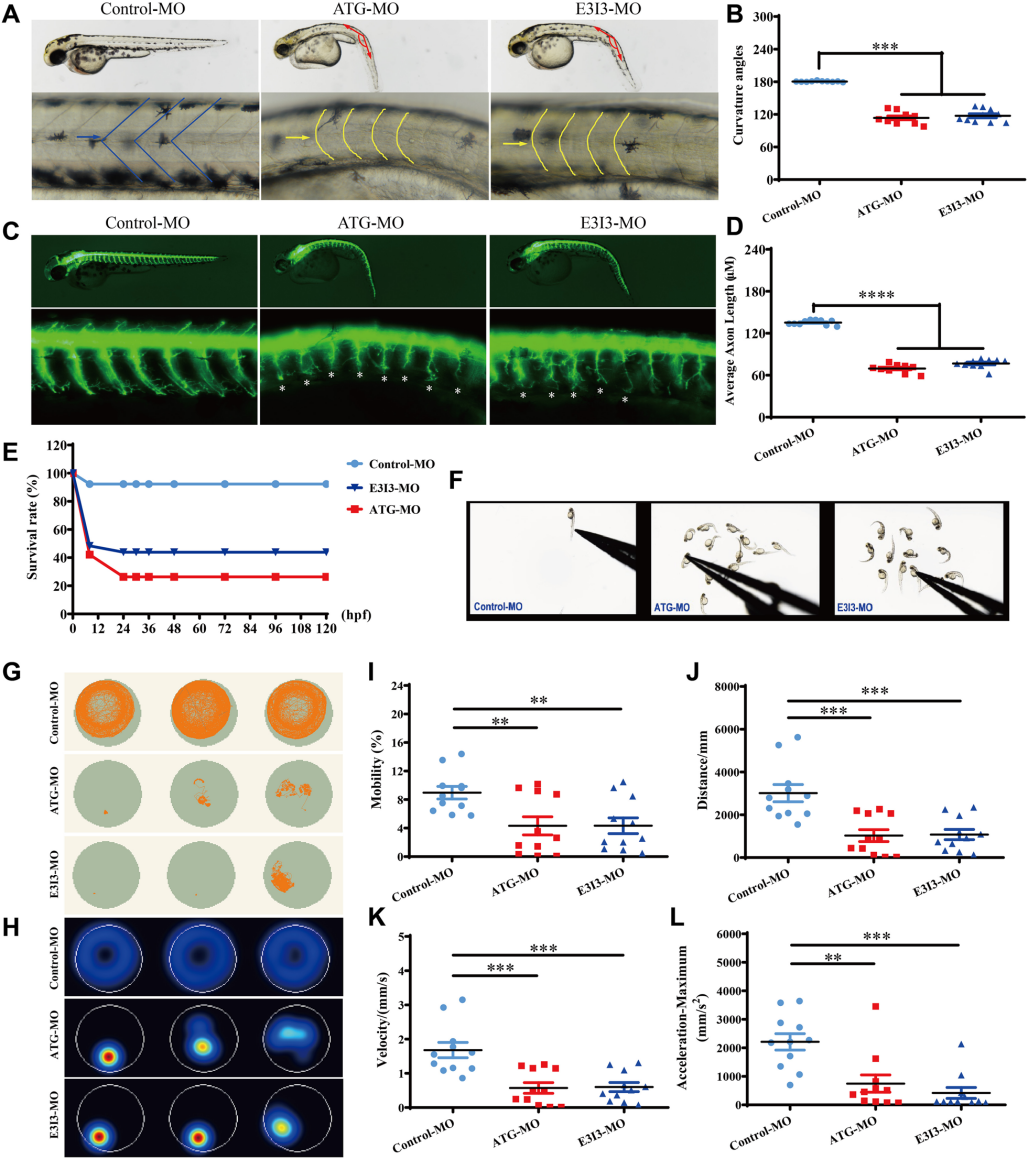
Locomotor capacity is reduced in *fars2* morphants. **A–B** Gross morphology of Tg (hb9:eGFP) zebrafish embryos at 54-hpf. hpf, hours post fertilization. Compared with control-MO, *fars2* morphants present a curved body axis (blue dotted line) and U-shaped somites (yellow line and arrow). Quantification of the average curvature angle (mean ± S.E.M; One-way ANOVA with Bonferroni posthoc test, ***P < 0.001; *n* = 10). **C** Gross morphology of Tg (hb9:eGFP) zebrafish embryos at 54-hpf. Red lines depict the definition of curvature angle, asterisk indicates impaired motor axon. **D** Quantification of the average motor axon length reveals a decrease in *fars2* morphants (mean ± S.E.M; One-way ANOVA with Bonferroni posthoc test, ****P < 0.0001; *n* = 10). **E** Time-course of percent survival in control vs. *fars2* morphants for 5 days. *n* = 100. **F** The stereotypic escape response, normal in control larvae but highly reduced or absent in *fars2* morphants. **G**–**H** Digital tracks and heat-map image in larvae from control-MO and *fars2*-MO injected groups at 5 dpf. dpf, days post fertilization. *n* = 10. **I**–**L** Total movement distance, velocity, mobility and maximum acceleration in *fars2*-MO group are significantly lower than those in the control-MO injected groups (mean ± S.E.M; One-way ANOVA with Bonferroni posthoc test, **P < 0.01; ***P < 0.001; *n* = 11).

Zebrafish model was made to obtain behavioral data that correlated with spastic paraplegia [40]. We investigated *fars2* expression during embryonic development in zebrafish by qRT-PCR. *fars2* is expressed early in the zebrafish embryo, and its expression increases between 14-hpf and 24-hpf (Additional file 1: Fig. S6A). *fars2-*knockdown zebrafish were obtained by microinjecting specific morpholino (MO) antisense oligonucleotides into fertilized one-cell stage embryos, and hb9:eGFP transgenic lines were used to study motor neurons and axons. qRT-PCR was used to confirm the effectiveness of *fars2* knockdown (Additional file 1: Fig. S6B and C). At 54 h post fertilization (hpf), both strains of *fars2*-knockdown zebrafish present curved bodies and U-shaped somites (Fig. 7A and B). Motor axon lengths were significantly decreased in *fars2* morphants, indicating impaired motor axon growth due to *fars2* deficiency (Fig. 7C and D). What’s more, early *fars2* deficiency during the embryonic period caused lethality. We calculated the survival rate of *fars2*-deficient zebrafish and discovered a significant decrease within 24-hpf, leaving 20–50% of surviving zebrafish (Fig. 7E).

Locomotor behavioral tests were then performed on 54-hpf larvae. In *fars2* morphants, the stereotypic escape response, which is normal in control larvae, was greatly reduced or absent (Fig. 7F; Additional file 2: movie S1). The digital tracks and corresponding heat-maps were recorded to further evaluate the motor capacity of the fish (Fig. 7G and H). The total movement distance, velocity, mobility, and maximum acceleration in the *fars2*-MO group were significantly lower than those of the control-MO injected groups, suggesting a phenotype similar to that of patients with HSP (Fig. 7I-L).

### **Potential downstream pathways during neuronal development in response to*Fars2*deficiency**

**Fig. 8:**
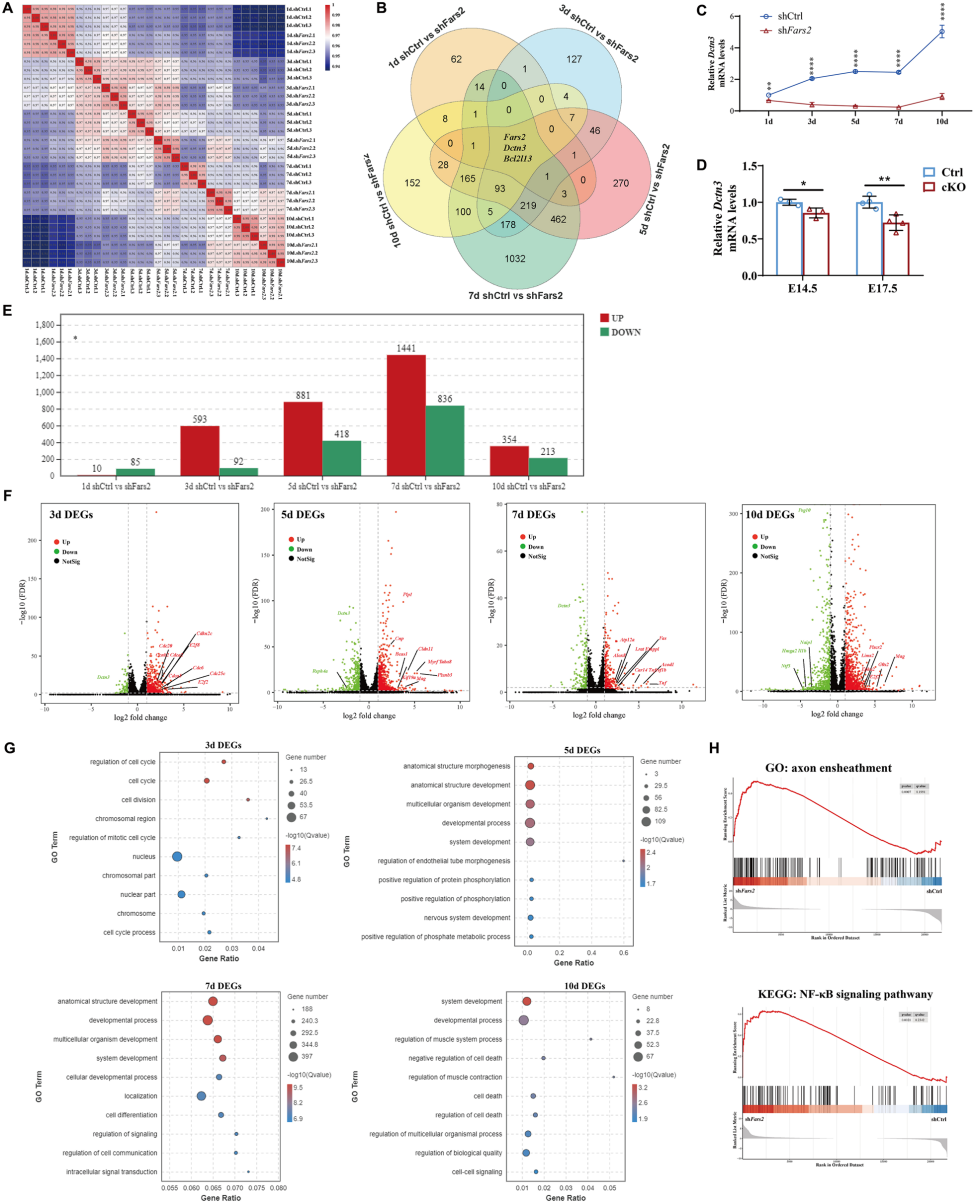
RNA-sequencing analysis of *Fars2* knockdown effect in mouse neurons in vitro. **A** Pearson correlation value of gene expression between samples based on RNA-seq. Each row and column of the figure both represent samples, the number and the square color below represent the correlation value between pair of samples. The more two samples are similar with each other, the closer the correlation value is to 1. **B** Venn diagram showing the distribution of unique and common DEGs among comparisons at different culture days. **C** qRT-PCR-based validation of *Dctn3* expression in primary cultured neurons at different time points. (mean ± SD; two-tailed unpaired t-Test, **P < 0.01; ****P < 0.0001; *n* = 3). **D** qRT-PCR-based validation of *Dctn3* expression in mouse cortex at different embryonic day. (mean ± SD; two-tailed unpaired t-Test, *P < 0.05; **P < 0.01; *n* = 3-4). **E** DEGs numbers in sh*Fars2* vs shCtrl group at 1, 3, 5, 7, 10 days post-infection (*n* = 3 from different littermates for each group). Adjust *P* < 0.05 and |logFC|≥ 2 were set as the cut-off criteria. **F** Volcano plot of the DEGs in sh*Fars2* vs shCtrl group at 3, 5,7,10 days after infection. Red dots represent up-regulated genes, green dots represent down-regulated genes, black dots represent genes with no significantly changes. **G** GO term enrichment analysis of DEGs between the *Fars2-*knockdown group versus the control group at 3, 5, 7, 10 days post-infection. The *P*-value has undergone FDR Correction (FDR ≤ 0.05). Gradation of dot color represents statistical significance and the size represents the number of implicated genes. **H** GSEA of primary cultured neuron transcriptomes revealed that genes involved in axon ensheathment and NF-κB signaling pathway are respectively upregulated at 7th and 10th day after infection in *Fars2* knocked-down group

To identify the molecular mechanisms and the dynamic changes underlying the neurodegenerative phenotypes caused by *Fars2* deficiency, we performed RNA-seq analysis at 1,3,5,7, and 10 days after infection using mouse primary cultured neurons. The Pearson correlation value between each sample was calculated based on the fragments per kilobase of exon model per million mapped fragments (FPKM) value of all the genes. The results showed that the correlation coefficients of samples within each group were all close to 1, indicating good biological repetition effect of samples within groups. The correlation coefficients between groups at different time points showed that the Pearson value of 1 d sh*Fars2* vs. 3 d sh*Fars2* were slightly higher than that of 1 d shCtrl vs. 3 d shCtrl. Similarly, the value of 3 d sh*Fars2* vs. 5 d sh*Fars2*, 5 d sh*Fars2* vs. 7 d sh*Fars2* and 7 d sh*Fars2* vs. 10 d sh*Fars2* was also slightly higher than that of 3 d shCtrl vs. 5 d shCtrl. 5 d shCtrl vs. 7 d shCtrl and 7 d shCtrl vs. 10 d shCtrl (Fig. 8A). These results suggest that the overall developmental process of sh*Fars2* neurons is slower than that of shCtrl neurons, which is consistent with our previously observation in primary cultured neurons and the developmental delay manifestation in human patients.

In order to explore the interaction between sh*Fars2* and shCtrl group differentially expressed gene sets at different developmental stages of neurons, we drew Venn plots for DEGs at each time point. The results showed that there were specific DEGs at each time point, while overlapped DEGs also existed. In addition to the *Fars2* gene, *Dctn3* and *Bcl2l13* have different expression pattern in *Fars2*-deficient samples at each neuronal development period (Fig. 8B). qRT-PCR validated that *Dctn3* decreased immediately after *Fars2* knockdown both in vitro and in vivo, and kept transcribing at lower level in comparison to control neurons during the whole developmental process (Fig. 8C and D).

To further explore the signal transduction alteration within *Fars2*-knockdown neurons at different developmental stages. We calculated the number of DEGs of sh*Fars2* vs. shCtrl group of 3 , 5 , 7  and 10 days spost-infection. The number increased steadily along with cultured time, and peaked on the seventh day after infection; 1441 up-regulated transcripts and 836 down-regulated transcripts met the statistical significance threshold (*p* < 0.05, fold change > 1.5 or 0.67), and then the change began to converge (Fig. 8E). Volcano plot displayed that the expression of some cell cycle regulation related genes such as *Cdc* family and *Cdkn* family were up-regulated in sh*Fars2* neurons at 3 d post-infection. At the stage of neurite extension on day-5, genes related to microtubule formation and axon development, such as *Tuba8* and *Kif19a*, as well as genes related to cell adhesion, such as *Mag* and *Cldn11*, were significantly up-regulated in sh*Fars2* group. While the expression level of cilia movement related gene, Rsph4a, decreased significantly, which may affect the growth of neuronal processes. On day-7, the expressions of some apoptosis-related genes including *Naip1* and *Peg10* began to decrease, the inhibition of *Peg10* was reported to activate apoptosis [41], which was consistent with the increase of apoptosis level of sh*Fars2* neurons on day-8. According to our previous results, the function of mitochondria in sh*Fars2* group neurons were severely damaged at 10 days post-infection. Transcriptome results showed that *Atp12a*, a gene related to the maintenance of membrane electrochemical gradient, was up-regulated in sh*Fars2* group neurons, and the expression level of a lipid metabolism related gene, *Alox8*, and a carbon dioxide transfer related gene, *Car14*, both up-regulated, which might contribute to regulating intracellular pH value (Fig. 8F). GO enrichment analysis showed the DEGs of each time points were enriched in different basic biological processes. On day 3, DEGs were mainly enriched in the pathways related to cell cycle regulation; On day 5, the enriched pathways were mainly related to cell morphology and development. On day 7, in addition to development-related pathways, pathways related to intercellular signal transduction were also significantly enriched. On day 10, several cell death regulation pathways were enriched (Fig. 8G). GSEA of primary cultured neuron transcriptomes revealed that genes involved in axon ensheathment are upregulated in *Fars2* knocked-down group at 7th day after infection, and genes involved in NF-κB signaling pathway are upregulated in *Fars2* knocked-down neurons at 10th day after infection (Fig. 8H).

## Discussion

Mutations in genes that encode mitochondrial aminoacyl-tRNA synthetase are increasingly linked to human neurodegenerative diseases. In a previous study, we discovered p.D142Y, a novel *FARS2* mutation that causes pure type HSP. In the present study, we reviewed all *FARS2* mutations associated with human disease and discovered a high degree of heterogeneity in the onset of symptoms and clinical outcomes in individuals with different mutation sites and types. What’s more, a general loss of enzymatic function mechanism was implied in human cases. Establishing reliable in vitro and in vivo models with *FARS2* deficiency will allow for the development of formal diagnostic criteria for *FARS2* deficiency, and the promotion of further research into its associated pathogenic mechanism. And in this study, we mainly discuss the effect of great loss of FARS2 canonical function in nerous systems.

Magnetic resonance imaging (MRI) in patients with *FARS2* mutations reveals various degrees of reduced cerebral white matter volumes, a common feature within brain structures [19]. White matter is composed of growing axon and dendrites and serves an important role in providing structural connectivity between gray matter regions via the formation and organization of neural networks, and further facilitates neurobehavioral operations [42]. The process of neurite outgrowth is energy-demanding. Mitochondrial dysfunction may therefore produce abnormalities in these neural processes, resulting in the onset and/or progression of neurodegenerative diseases such as epilepsy and Alzheimer’s disease [43, 44]. In the present study, mouse neurons deficient for *Fars2* exhibited highly-impaired neurite outgrowth, preventing neuron network formation. This is consistent with white matter loss in the brain of patients with *FARS2* mutations. The strong degeneration of motor neurons in zebrafish, and excessive neuronal death within the motor cortex in *Fars2*-deficient mouse models, clarified the spastic paraplegia phenotype observed in human patients with *FARS2* mutations. This is further supported by the observed reduction in motor ability in the surviving zebrafish.

In early-onset epileptic encephalopathy patients with *FARS2* mutations, global brain atrophy, and cortical atrophy in particular, is a general MRI change in the later course of the disease. The present data show that *Fars2-*deficient mouse embryos have a thinner cortex and an enlarged ventricle; these correspond to the neural atrophy observed in human brain. Subsequent experiments suggested that progressive neuronal apoptosis is a response to cortical tissue thinning that eventually leads to brain atrophy. This biological response is a common occurrence in many late-stage neurometabolic disorders.

Apart from changes in MRI, most *FARS2-*mutant patients exhibited elevated lactate levels; this may signal anaerobic metabolism caused by mitochondrial dysfunction. In the present study, mitochondrial function was comprehensively examined in animal models and in vitro. *Fars2-*deficient mouse embryos had a lower mitochondrial inner membrane potential, indicating lower mitochondrial activity. Furthermore, the strong reduction in ATP levels suggested that mitochondrial bioenergetic functions were reduced. ROS levels are a sign of oxidant-antioxidant homeostasis in organisms because they are a byproduct of mitochondrial oxidative metabolism [45]. Low expression of *Fars2 in vitro* and in vivo leads to elevated levels of ROS. Excessive ROS availability contributes to neuronal cell death via stimulation of those biochemical pathways associated with oxidative stress and cellular apoptosis [46, 47]. The NAD^+^/NADH dinucleotide pair maintains a proper NAD^+^/NADH ratio under physiological conditions by driving a wide range of reduction–oxidation (redox) reactions and inhibiting excessive ROS generation [48]. Total NAD of the mitochondria pool, however, were reduced in *Fars2*-deficient tissues and cells; this suggests an imbalance between oxidant and antioxidant levels in organisms deficient for *Fars2*.

Given the classic aminoacylation function of mtPheRS, disorganized mitochondria translation machinery is a prime suspect in *Fars2* deficiency-induced mitochondrial dysfunction. The present data reveal that *Fars2* deficiency affects the expression levels of OXPHOS-subunit protein and subsequently the stable levels of OXPHO complexes. In the mouse brain, *Fars2*-KO reduced mitochondrial CI, CIII, and CIV; *Fars2*-knockdown in primary neurons in culture, however, only affected CI of the electron transport chain. This phenotypic heterogeneity was also observed in patients with *FARS2* mutations, and could be attributed to varying degrees of aminoacylation function loss in different organisms.

Results in this study indicate that CI is more vulnerable to *Fars2* deficiency. It is reported that CI deficiency is the most common OXPHOS disorder and may be due to the complicated and multifaceted assembly process required by CI [49, 50]. As the first enzyme in OXPHOS, CI oxidizes NADH into NAD^+^ and drives proton translocation across the mitochondrial inner membrane together with CIII and CIV; this forms the mitochondrial membrane potential that then promotes ATP generation by CV [51]. The present data show that expression of CI level was severely reduced in both in vitro and *in vivo Fars2-*deficient environments, leading to the reduced conversion of NADH to NAD^+^ and limited proton translocation. Reduced electron transfer produces mitochondrial depolarization, and the release of ROS ultimately diminishes ATP production.

With the exception of decreased CI, CIII, and CIV, the slight enhancement of CII and CV and the general upregulation of subunit mRNA levels highlight a compensatory mechanism against dysfunctional mitochondrial translation. Furthermore, transcriptome sequencing analysis revealed that the *Fars2*-defect induced neuronal dysplasia may closely related to the activation of axon sheathing process and NF- κB signaling pathway during neuron development. The myelin sheath is critical for increasing neural processing speed and efficiency [52], whose activation might be a compensatory rescue pathway in respond to *Fars2* knockdown in neurons. NF-κB pathway activation is reported to be required for the initiation of apoptosis, and also be a key mediator of neuroinflammation in various neurodegeneration disease including Alzheimer’s Disease and Parkinson’s Disease [53, 54].

Although several researches have suggested non-canonical function, such as post-transcriptional regulation of mRNA expression, in some aaRSs [55]. No evidence for FARS2 function other than mitochondrial translation activity has been shown yet. While in this study, transcriptome analysis reveals a potential downstream molecule of *Fars2* deficiency, *Dctn3*. *Dctn3* is a subunit of Dynactin, which is a multisubunit protein complex required for activating dynein and regulating retrogradely transports cargo along microtubules [56]. Dysregulation of retrograde axon transport is considered to be a pivotal pathogeny of motor neuron degeneration disease [57]. These evidence suggests that in addition to mitochondrial dysfunction, disruption of retrograde axon transport induced by *Dctn3* reduction might be another possible pathogenesis of *Fars2*-related neuropathy.

A limitation of this study is that we only focus on *Fars2*-deficient neurons and their fate of apoptosis. However, apoptotic cells were observed both in dorsal and ventral cortex region of E 14.5 cKO mouse embryos during cortical neurogenesis, which suggests that neural progenitors might also be affected. Whether and how *Fars2* deficiency affect the proliferation and differentiation of neural progenitors would be an important aspect to study in the future.

## Conclusions

**Fig. 9:**
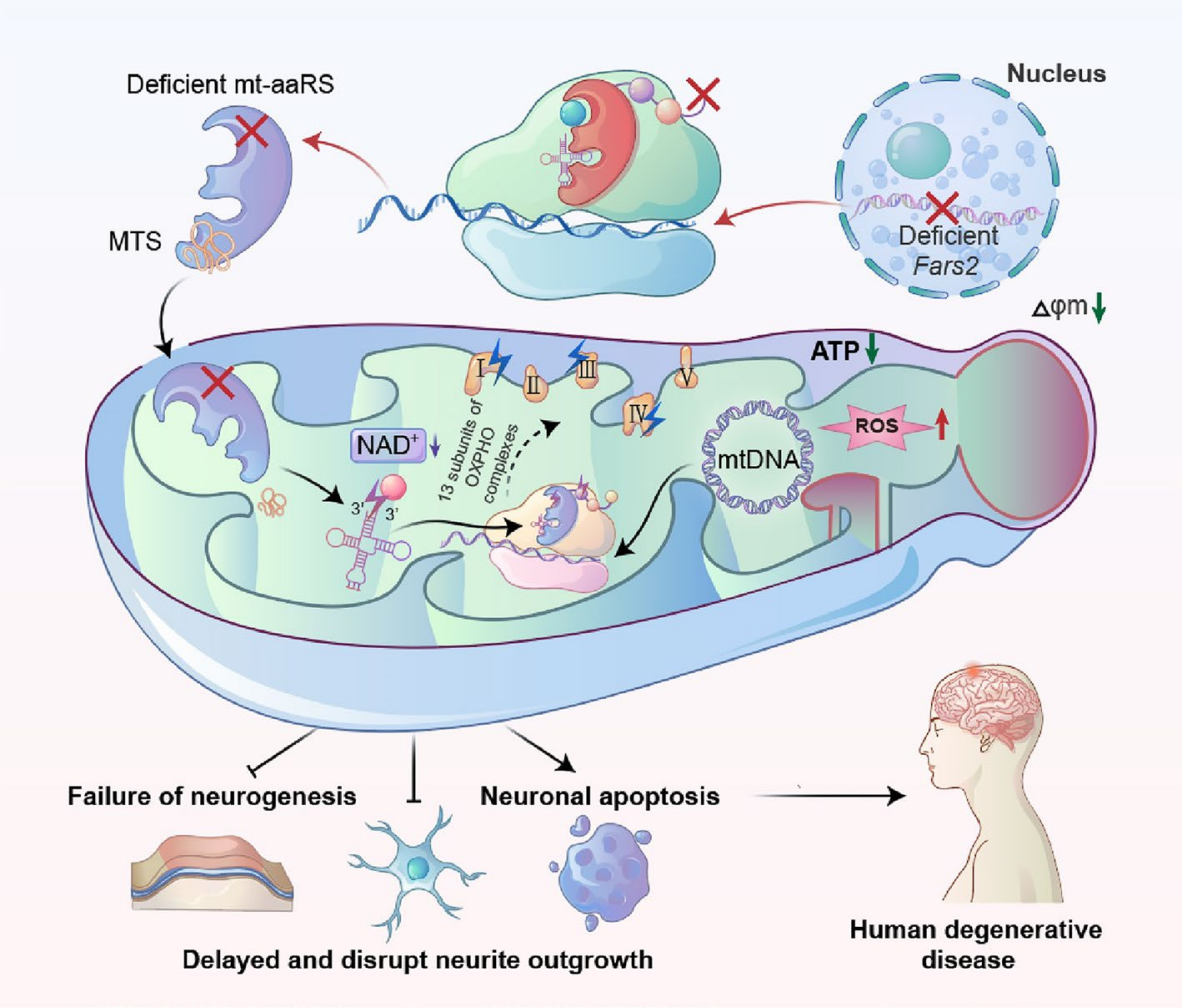
Neuropathy-associated *Fars2* deficiency affects neuronal development, potentiates neuronal apoptosis by impairing mitochondrial function. An overview of the mechanisms by which *Fars2* deficiency disrupts mitochondrial translation, which leads to neuronal apoptosis and possibly neurodegenerative diseases

To sum up, we have successfully established mouse and zebrafish models of *Fars2* deficiency that mimic the neuropathy and metabolism changes seen in human patients. We found that dysfunction caused by disruption to mitochondrial translation leads to neuronal apoptosis and possibly neurodegenerative diseases. Taken together, our study emphasizes the critical role of *Fars2* in mitochondrial function and its pathological consequences for neuronal development and maintenance (Fig. 9).

## Materials and methods

Animal models were established under technical and facility support of Shanghai Model Organisms Center. All experiments were conducted with ethics approval from the Air Force Medical University under regulations of the Guide for the Care and Use of Laboratory Animals published by the National Institutes of Health.

### **Global*Fars2*-mutant and*Fars2*-KO mice**

Mice with whole-body p.D142Y mutant and KO were generated by using CRISPR/Cas 9 technology. In brief, Cas9 mRNA, guide RNA were obtained by in vitro transcription. Oligo donor DNA with mutant point was synthesized. The F0 p.D142Y mice was generated by microinjecting Cas9 mRNA, guide RNA (GGGACAACTATTACTTGAAT) and donor DNA (GACCAGCTTCCTCCAGTGGTCACCACCTGGCAGAACTTTGATAGCCTGCTAATCCCAGCTGACCACCCCAGCAGGAAAAAGGGGTACAACTATTACTTGAATCGCGCACACATGCTGAGAGCACACACATCAGCGCATCAGTGGGACTTGCTGCATGCGGGACTTAATGCCTTCCTTGTG) into the oosperm of C57BL/6J mice. The F0 KO mice was generated by microinjecting Cas9 mRNA, guideRNA (GGGACAACTATTACTTGAAT) into the oosperm of C57BL/6J mice. The genotypes of offspring mice were determined by PCR and Sanger sequencing.

### **Conditional*Fars2*-KO mice**

LoxP were inserted into *Fars2* of C57BL/6J mice by injecting Cas9 mRNA, guide RNA (gRNA#1: AGCAAGCTCTGAGCTACCCAGGG; gRNA#2: GGACAAGATGCTTCACAATATGG) and donor vector into the oosperm to obtain *Fars2* ^fl/fl^ mice. CNS-specific *Fars2*-deficient mice were generated by mating *Fars2* ^fl/fl^ with transgenic mice containing Nestin driven Cre recombinase [58]. The Nestin-Cre mouse was purchased from Shanghai Model Organisms (China). The genotypes of offspring mice were determined by PCR and Nucleic acid gel electrophoresis.

### MO-Zebrafish

Adult wild-type AB strain zebrafish pairs were mated under 28.5 ℃ on a 14 h light / 10 h dark cycle in fish water (0.2% Instant Ocean Salt in deionized water). An average of 200–300 embryos were generated. The embryos were washed and staged according to the previous study [59]. The hb9:eGFP transgenic line was established according to the previous described [60]. *fars2* translation-blocking morpholino (ATG-MO), splice-blocking morpholino (E3I3-MO) and standard control morpholino (MO) were designed and produced by Gene Tools, LLC (http://www.gene-tools.com/). The MO sequences were listed in Additional file 1: Table S1. MOs were injected into fertilized one-cell stage embryos according to standard protocols [61]. The amount of the MOs used for injection was as follows: 4ng Control-MO and ATG-MO, 8ng E3I3-MO were used for injection per embryo. RT-PCR was performed to confirm the efficacy of the E3I3-MO. ef1α was used as the internal control. The sequences of the primers were listed in Additional file 1: Table S2. The zebrafish facility at Shanghai Model Organisms Center is accredited by the Association for Assessment and Accreditation of Laboratory Animal Care International.

### Zebrafish spinal motor neurons studies

To evaluate spinal motor neurons formation in zebrafish, fertilized one-cell hb9:eGFP transgenic lines embryos were injected with 4ng control-MO, 4ng *fars2*-ATG-MO or 8ng *fars2*-E3I3-MO. At 54-hpf, embryos were dechorionated, anesthetized with 0.016% tricaine methanesulfonate (Sigma-Aldrich). Zebrafish were then oriented on lateral side or dorsal side, and mounted with 3% methylcellulose in a depression slide for observation by fluorescence microscopy. The phenotypes of spinal motor neurons were analyzed.

### Stereotypic escape response assays

54-hour-old living zebrafish larvae were dechorionated manually at least 3 h before the experiment. To evaluate the escape response, fish were touched with the tip of a fine needle for at least 2 times at the dorsal tip of the tail or trunk. An escape response in which the fish did not move a distance of at least 3 times its own body length was considered as reduced.

### Behavioral analysis

At 5-dpf, the larvae were divided into three groups: control-MO, *fars2*-ATG-MO and *fars2*-E3I3-MO. Larvae from each group were collected, cleaned and placed in 96-well plates. Each well contained 0.2 ml of fish water and one larva, and ten larvae were in each group. Behavioral tests were performed as following: the larvae were allowed to acclimate for 15 min before locomotion monitoring [62]. Next, the larvae were allowed to freely explore the aquarium for 30 min. A camera positioned above the plate was used for movement tracking. All digital tracks were analyzed by Ethovision XT software (Noldus Information Technology, Wageningen, Netherlands), and a minimum movement distance of 0.2 mm filtered out system noise. Four parameters, including the total movement distance, velocity, mobility and maximum acceleration were analyzed. Embryos and larvae were analyzed with Nikon SMZ18 Fluorescence microscope and subsequently photographed with digital cameras. A subset of images was adjusted for levels, brightness, contrast, hue and saturation with Adobe Photoshop 7.0 software (Adobe, San Jose, California) to optimally visualize the expression patterns. Quantitative image analyses processed using image based morphometric analysis (NIS-Elements D4.6, Japan) and ImageJ software (U.S. National Institutes of Health, Bethesda, MD, USA).

### Brain separation and section preparation

Pregnant mice of proper days were deeply anesthetized with 10% chloral hydrate and the embryos were extracted. Fresh brain of each embryo were isolated in precooled PBS and then fixed in 4% paraformaldehyde overnight. For paraffin sections, the tissue was dehydrated in Gradient alcohol and embedded in paraffin. Coronal sections were made at a 3 μm thickness with a paraffin slicing machine (Leica, RM2125RTS). For cryostat sections, brain was placed in 30% sucrose until sink to the bottom and and embedded in Tissue Freezing Medium (Leica, 03819110). sectioned at a 12 μm thickness with a freezing microtome (Leica, CM1950).

### Primary neuron culturing and lentivirus infection

Cultures of cortical neurons were prepared from embryonic day 14–16 mouse brains as described previously with some modifications.[63] In brief, embryo cortex was isolated in pre-cooled HBSS (Gibco, 14,175,095). After removing the meninges, tissue was cut into pieces and transferred into Neurobasal Medium (Gibco, A3582901) supplemented with 2% B27 (Gibco, 17,504,044), 1% L-Glutamine (Gibco, A2916801). Blowed the tissue pieces into suspension using a pipettor. Filtrate the suspension by using a 70 μm Cell Strainers (FALCON, 352,350) to obtain single-cell suspension and then seed into dishes pre-coated by Poly-L-Lysine (Sigma, P1399). After 1 days in vitro, rLV-U6-sh*Fars2*-CMV-mcherry-WPRE and rLV-U6-shNC(scramble)-CMV-mcherry-WPRE (Brainvta Company, China) were added into the dishes. shRNA sequences were listed in Additional file 1: Table S3. The culture medium was changed after 12 hours’ infection. Half amount of medium was changed every 2 days in the following cultivation.

### Immunofluorescence

Primary cultured neurons were seeded on round glass coverslip in 24-well plates at a density of 2 × 10^5^ per well. After infection, cells were fixed and proceed to regular immunofluorescence at indicated days. The following antibodies were used: MAP2 (Abcam, ab11267, 1:1000), Neuron-specific β- Tubulin (TUJ1) antibody (R&D, MAB1195-SP, 1:500). Secondary Antibodies was purchased from Jackson ImmunoResearch: Alexa Fluor® 488-conjugated AffiniPure Goat Anti-mouse IgG (H + L) (115-545-003).

### Hematoxylin and eosin staining

Embryos at E 8.0 were isolated from the pregnant mice after cross-mating and fixed with 4% paraformaldehyde, dehydration in 30% sucrose and embedded in Tissue Freezing Medium. Cryostat sections with 12 μm thickness were made by a freezing microtome and stained with Hematoxylin and Eosin Staining Kit (Beyotime, C0105S). The images were detected under a light microscopy.

### Nissl staining

Paraffin sections of embryo brain were prepared and incubated for 45 s in Nissl solution (Beyotime, C0117) followed by dehydration with different ethanol concentrations as described in the protocol of the instructions.

### TUNEL assay

Apoptotic cells were detected by using Fluorescein (FITC) Tunel Cell Apoptosis Detection Kit (Servicebio, G1501) according to the instruction. In brief, cells coverslips or mouse brain sections were fixed with 4% paraformaldehyde, rinsed with PBS, and then permeabilized by 0.1% Triton X-100. The TUNEL reaction mixture was used for FITC end-labeling the fragmented DNA of the apoptotic cells. The cell nuclei were stained with 2 mg/ml DAPI (Invitrogen, D1306). The FITC-labeled TUNEL-positive cells were imaged under a fluorescence microscope.

### Detection of cellular ATP levels

Cellular ATP levels were measured using a firefly luciferasebased ATP assay kit (Beyotime, S0026) according to the manufacturer’s instructions. Briefly, cortical tissue or harvested cultured cells were lysed with a lysis buffer, and centrifuged at 12,000 × g for 5 min. 20 µl of each supernatant or standard substance was mixed with 100 µl luciferase reagent. Luminance (RLU) was measured by a multimode microplate reader (Tecan Spark). Standard curves were generated and the protein concentration of each sample was measured by BCA protein assay. Total ATP levels were expressed as pmol/ng protein.

### Measurement of reactive oxygen species

In brief, adherent neurons or cell suspension from cortical tissue were incubated with 6-Carboxy-2′, 7′-dichlorodihydrofluorescein diacetate (DCFH-DA) (Beyotime, S0033S) at a final concentration of 10 mM for 20 min and washed 3 times with PBS. And then ROS generation was measured by the fluorescence intensity with excitation and emission settings at 488 and 525 nm.

### Measurement of NAD level

NAD_total_ levels and NAD^+^/NADH were determined using NAD^+^/NADH assay kit with WST-8 (Beyotime, S0175) according to the manufacturer’s instructions. In brief, adherent neurons or cortical tissues were lysed with 400 µl of lysis buffer, and centrifuged at 12,000 ×g for 10 min. 90 µl of alcohol dehydrogenase was added to a 96-well plate. NAD_total_ levels were obtained by adding 20 µl of the supernatant or standard substance. And NADH levels were obtained by adding 20 µl of the suspension or standard substance after incubating at 60 °C for 30 min and was added to a 96-well plate. Subsequently, 10 µl of chromogenic solution was added to the plate and the mixture was incubated at 37 °C for 30 min. The absorbance values were measured at 450 nm and analyzed on a multimode microplate reader (Tecan Spark). Standard curve was generated and the protein concentration of each sample was measured by BCA protein assay. The amount of NAD^+^ was derived by subtracting NADH from NAD_total_.

### Transmission electron microscopy detection

Primary neurons and PC12 Cells were respectively digested using Neuronal Isolation Enzyme (with papain) (Thermo 88,285) and 0.25% Trypsin-EDTA (Gibco, 25,200,072), and then centrifuged at 800 rpm for 10 min to obtain cell pellet. Cell pellets and cortex blocks were fixed in 2.5% glutaraldehyde for 24 h and then post-fixed in 2% osmium tetroxide for 1 h. After dehydration, the specimens were embedded in epoxide resin. Ultrathin sections were made and stained with uranyl acetate and lead citrate, images were captured at a magnification of 20,000 × and 300,000 × on a Zeiss electron microscope.

### Measurement of mitochondrial membrane potential

JC-1 kit (Beyotime, C2006) was employed to estimate mitochondrial depolarization in cortical tissue and PC12 cell. Briefly, cell suspension from cortical tissue or adherent cells after indicated treatments were incubated with an equal volume of JC-1 staining solution (5 µg/ml) at 37 ℃ for 20 min and rinsed twice with PBS. Mitochondrial membrane potentials were monitored by determining the relative amounts of dual emissions from mitochondrial JC-1 monomers or aggregates indicated by the green/red fluorescence intensity ratio using FAC Scalibur flow cytometer (BD).

### Preparation of mitochondrial and cytosolic fractions

Mitochondrial and cytosolic fractions were isolated using a commercially available cytosol/mitochondria fractionation kit according to the manufacturer’s protocol (Beyotime, C3606).

### Western blotting

Cortical and neuron cell were lysed with the RIPA buffer (Beyotime, P0013B). Protein extracts were analyzed by 10% SDS-PAGE and blotted onto PVDF membrane. The following primary antibodies were used at indicated concentrations: Total OXPHOS rodent antibody (Abcam, ab110413, 1:250); ND1 antibody (Abcam, ab181848, 1:10000); CO2 antibody (Abcam, ab198286, 1:1000); CYTB antibody (LifeSpan BioSciences, 197,737, 1:500); TOMM20 antibody (Abcam, ab186735, 1:5000); Caspase-3 antibody (Cell Signaling Technology, 9662, 1:1000); Cleaved Caspase-3 antibody (Cell Signaling Technology, 9661, 1:1000); β-actin antibody (Sigma, A1978, 1:3000); FARS2 antibody (Invitrogen, PA5-53738, 1:1000). All secondary antibodies were purchased from ZhuangzhiBio (anti-mouse, 1: 8000; anti-rabbit, 1:8000) Western blotting quantification was performed using the ImageJ software.

### Quantitative real-time PCR

Total RNA was isolated from tissue or cultured cells using Multisource Total RNA Miniprep Kit (Axygen, 365), and then used for cDNA synthesis (PrimeScript™RT Master Mix, Takara, RR036Q) and amplification by real-time PCR according to the manufacturer’s instructions (SYBR Premix Ex Taq™ II, Takara, RR820A). Relative gene expression quantification was based on the comparative threshold cycle method (2^−ΔΔCt^) using β-actin as endogenous control gene. The primer sequences were provided in Additional file 1: Table S2.

### RNA extraction, library construction and transcriptomic sequencing

Total RNA from primary cultured neurons after 1,3,5,7,10 days of infection was extracted using Multisource Total RNA Miniprep Kit (Axygen, 365). RNA quality was performed using Agilent 2100 Bioanalyzer (Agilent Technologies, USA) and checked using RNase free agarose gel electrophoresis. After qualification, total mRNA was enriched by Oligo (dT) beads (Epicentre, USA). RNA was fragmented into short fragments and reverse transcript into cDNA with random primers. Second-strand cDNA were synthesized and the cDNA fragments were purified with QiaQuick PCR extraction kit (Qiagen, Netherlands), end repaired, poly A added, and ligated to Illumina sequencing adapters. Selecting ligation products with proper size by PCR and agarose gel electrophoresis, and then sequencing was performed by Gene Denovo Biotechnology Co. (Guangzhou, China) on Illumina HiSeq2500.

### Bioinformatics analysis

The obtained reads were filtered by fastp (version 0.18.0) to get high quality clean reads without adapters or low-quality bases. An index of the reference genome was built, and paired-end clean reads were aligned to the mouse (mm10) genomes from UCSC using HISAT2 (v2.1.0). For RNAs differential expression analysis, reads were pseudo aligned to mouse (GENCODE GRCm38 release M16) transcriptomes using DESeq2 software between two different groups. Transcripts with log2 |fold change| > 1 and false discovery rate (FDR) < 0.05 were considered differentially expressed. Gene set enrichment analysis (GSEA), venn diagram, volcano Plot and gene ontology (GO) enrichment analysis were performed at Omicsmart online platform (https://www.omicsmart.com) based on the screened out differentially expressed genes (DEGs).

### Statistical analysis

Data were analyzed using GraphPad Prism 8 software. Statistical significance was calculated using student’s t test or ANOVA as appropriate. Data are presented as mean ± SD or SEM. Asterisks indicate the level of statistical significance, *P < 0.05, **P < 0.01, ***P < 0.001, ****P < 0.0001.

## Supplementary Information

**Additional file 1: Supplementary Methods. Figure S1.** Fars2 expression pattern during mouse embryonic development and in multiple organs of E 17 mouse embryos. **Figure S2.** Homozygous Fars2 knockout mouse cannot survive to born. **Figure S3.** Establishment of conditional neural-specific Fars2 knockout-mouse model.** Figure S4.** Establishment of Fars2-knockdown neurons in vitro using shFars2 lentivirus. **Figure S5.** Mitochondrial dysfunction was confirmed in PC12 cell line. **Figure S6.** Effectiveness of fars2 knockdown was confirmed by RT-PCR. **Table S1. **Table of Morpholino sequences. Table S2. Table of primer sequences in qPCR. **Table S3. **Table of shRNA sequences. **Table S4. **Table of siRNA sequences. and **Table S5. **Proportion of phenylalanine in 5 mitochondrial complexes in human, mouse, zebrafish and rat.

**Additional file 2: Movie S1.** Locomotor behavioral tests on 54-hpf larvae. The stereotypic escape response of fars2 morphant was greatly reduced or absents compared to control larvae.

## Data Availability

The datasets used and analysed during the current study are available from the corresponding author on reasonable request.

## Abbreviations

ATG-MO: Translation-blocking morpholino
CO2: Cytochrome c Oxidase Subunit 2
CYTB: Cytochrome b
CI-CV: Complex I-Complex V
DEGs: Differentially expressed genes
E: Embryonic day
E3I3- MO: Splice-blocking Morpholinos
FPKM: Fragments Per Kilobase of exon model per Million mapped fragments
GFP: Green Fluorescent Protein
GO: Gene Ontology
GSEA: Gene Set Enrichment Analysis
Hpf: hours post fertilization
HSP: Hereditary Spastic Paraplegia
KEGG: Kyoto Encyclopedia of Genes and Genomes
KO: Knockout
MAP2: Microtubule Associated Protein 2
MO: Morpholino
MRI: Magnetic resonance imaging
mt-aaRSs: mitochondrial aminoacyl-tRNA synthetases
mtPheRS: mitochondrial phenylalanyl-tRNA synthetase
mt-tRNAPhe: phenylalanine mitochondria tRNA
NAD: Nicotinamide Adenine Dinucleotide
ND1: NADH Dehydrogenase Subunit 1
OXPHOS: Oxidative Phosphorylation
ROS: Reactive Oxygen Species
shCtrl: scramble shRNA as a negative control
sh*Fars2*: *Fars2*-targetting shRNA
TOMM20: Translocase of Outer Mitochondrial Membrane 20
TUJ1: β- Tubulin
Δψm: Mitochondrial Membrane Potential
