# Supplementary material for: Neuropathy-associated Fars2 deficiency affects neuronal development and potentiates neuronal apoptosis by impairing mitochondrial function
Source: Cell Biosci. 2022 Jul 6;12:103. doi: 10.1186/s13578-022-00838-y (PMC9258231; doi:10.1186/s13578-022-00838-y)
Supplement: Supplementary file 1 — Additional file 1: Supplementary Methods. Figure S1. Fars2 expression pattern during mouse embryonic development and in multiple organs of E 17 mouse embryos. Figure S2. Homozygous Fars2 knockout mouse cannot survive to born. Figure S3. Establishment of conditional neural-specific Fars2 knockout-mouse model. Figure S4. Establishment of Fars2-knockdown neurons in vitro using shFars2 lentivirus. Figure S5. Mitochondrial dysfunction was confirmed in PC12 cell line. Figure S6. Effectiveness of fars2 knockdown was confirmed by RT-PCR. Table S1. Table of Morpholino sequences. Table S2. Table of primer sequences in qPCR. Table S3. Table of shRNA sequences. Table S4. Table of siRNA sequences. and Table S5. Proportion of phenylalanine in 5 mitochondrial complexes in human, mouse, zebrafish and rat. [file 13578_2022_838_MOESM1_ESM.pdf]

**Supplementary Materials for**

# Neuropathy-associated *Fars2* deficiency affects neuronal development and potentiates neuronal apoptosis by impairing mitochondrial function

Xihui Chen [1,2†], Fangfang Liu [3†], Bowen Li [1,2†], Yufeng Wang [1,2,4], Lijuan Yuan [1,2,5], Anan Yin [6], Qi Chen [1,2], Weihong Hu [1,2,4], Yan Yao [1,2,4], Mengjie Zhang [1,2,4], YuanMing Wu [1,2*], Kun Chen [7*]

**[†]Xihui Chen, Fangfang Liu and Bowen Li contributed equally to this work;**

***Correspondence to Yuanming Wu: wuym@fmmu.edu.cn and Kun Chen: chenkun@fmmu.edu.cn**

**Author affiliations:**

1 Department of Biochemistry and Molecular Biology, School of Basic Medicine, Air Force Medical University, Xi’an, Shaanxi, P.R. China.

2 Shaanxi Provincial Key Laboratory of Clinic Genetics, Air Force Medical University, Xi’an, Shaanxi, P.R. China.

3 Department of Neurobiology, School of Basic Medicine, Air Force Medical University, Xi'an, Shaanxi, P.R. China.

4 Medical Genetics, Yan’an University, Yan’an, Shaanxi, P.R. China.

5 Department of General Surgery, Tangdu Hospital, Air Force Medical University, Xi’an, Shaanxi, P.R. China.

6 Department of Neurosurgery, Xijing Institute of Clinical Neuroscience, and Department of Plastic surgery, Xijing Hospital, Air Force Medical University, Xi'an, Shaanxi, P.R. China.

7 Department of Anatomy, Histology and Embryology and K.K. Leung Brain Research Centre, School of Basic Medicine, Air Force Medical University, Xi’an, Shaanxi, P.R. China.

**Supplementary Methods**

**Cell line culture and siRNA transfection**

PC12 cells were cultured in DMEM supplemented with 10% FBS under 37 °C, 5% $CO_2$. Cells were transfected at 70% density using X-tremeGENE siRNA Transfection Reagent (Roche) according to the instrument. Non-specific control (si-Ctrl) and *Fars2*-specific siRNA (si-*Fars2*) was synthesized by Gene Pharma (China). siRNA sequences were listed in Supplementary Table 4. Briefly, PC12 cells were plated in a 3.5 cm dish and cultured overnight, 2 μg siRNA and 10 μl of X-tremeGENE Reagent were diluted in Opti-MEM (Gibco Cat. # 31985070) respectively. The dilution was mixed for 10 min at room temperature and then added into the cell dishes. The cells were harvested and analyzed at 24 h ,48 h and 72 h after transfection for specific measurements mentioned in this study.

**Annexin V-FITC/PI assay**

The mode of PC12 cell death was measured by annexin V and propidium iodide (PI) staining using FITC annexin V Apoptosis Detection Kit (BD Pharmingen). Briefly, cells after proper treatment were suspended in the binding buffer provided in the kit, and labeled with 5 μl of annexin V-FITC reagent and 5 μl of PI for 30 min at room temperature in the dark. The cells were then analyzed by FAC Scalibur flow cytometer (BD).

**Seahorse**

Seahorse XF24 extracellular flux analyzer (Agilent) and Seahorse XF Cell Mito Stress Test Kit (Agilent, 103015-100) was used to determining the oxygen consumption rate of PC12 cell. Cells were seeded at 40000/well and transfected with the corresponded siRNA. Prepare assay medium by supplementing Seahorse XF Base Medium with 1 mM pyruvate, 2 mM glutamine, and 10 mM glucose, pH 7.4. After 72 h transfection, the assay medium was added into the cell plate. 10 μM Oligomycin A, 20 μM carbonylcyanide m-chlorophenylhydrazone (FCCP), 5 μM Rotenone/antimycin A (Rot/AA) were added into different ports of the Seahorse cartridge. Protein concentration of each well was measured by BCA Protein assay. $O_2$ consumption rates (pmol/min) were measured and normalized.

**Supplementary Figures**

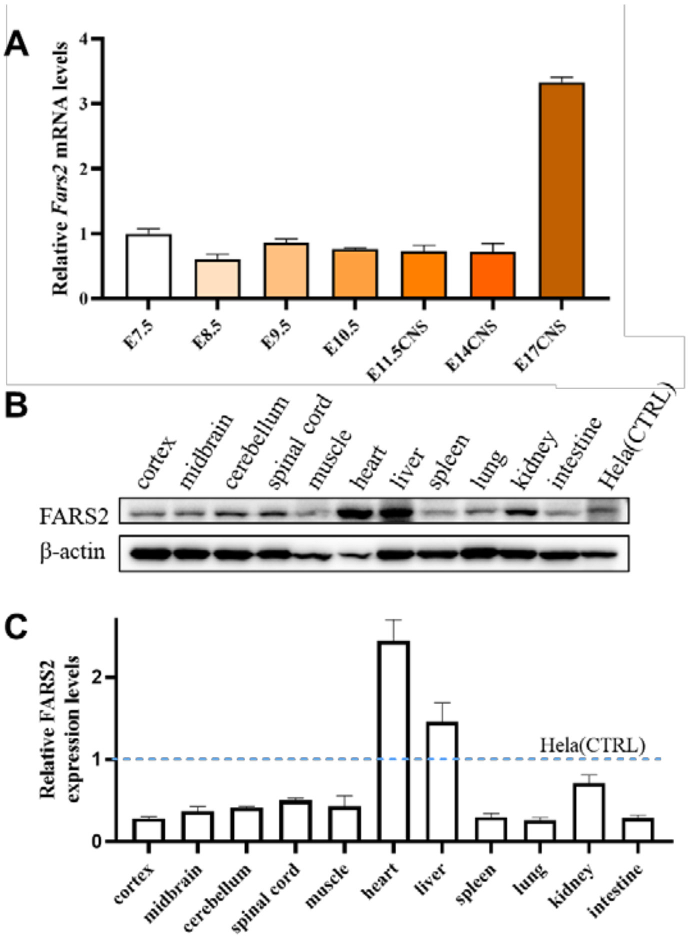


**Figure S1. *Fars2* expression pattern during mouse embryonic development and in multiple organs of E 17 mouse embryos.**

(A) *Fars2* mRNA level of whole mouse embryos at E7.5, E8.5, E9.5, E10.5 and CNS of E11.5, E14 and E17. (B) Western blot for FARS2 in the cortex, midbrain, cerebellum, spinal cord, muscle, heart, liver, spleen, lung, kidney and intestine of E 17 mouse embryos. Hela cell line was used as a positive control. (C) Quantification of B.

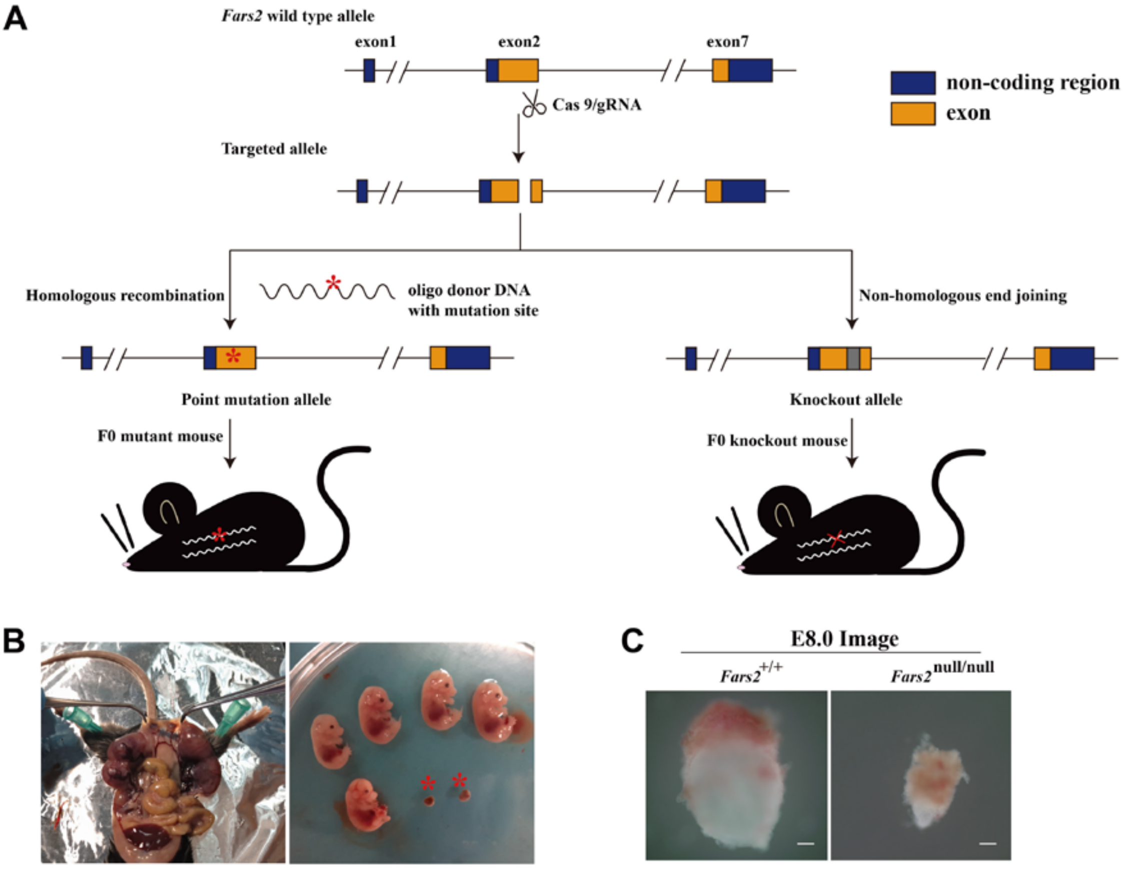


**Figure S2. Homozygous *Fars2* knockout mouse cannot survive to born.**

(A) Diagram of using CRISPR/Cas9 system to create heterozygous *Fars2* global mutant and knockout (KO) mice. (C) Photograph of embryos from *Fars2* $^{null/+}$ intercrossing at E 14.5. (C) Lateral view of wildtype and homozygous *Fars2* KO mice embryos at E 7.5-E 8.0. Scale bar: 0.25 mm.

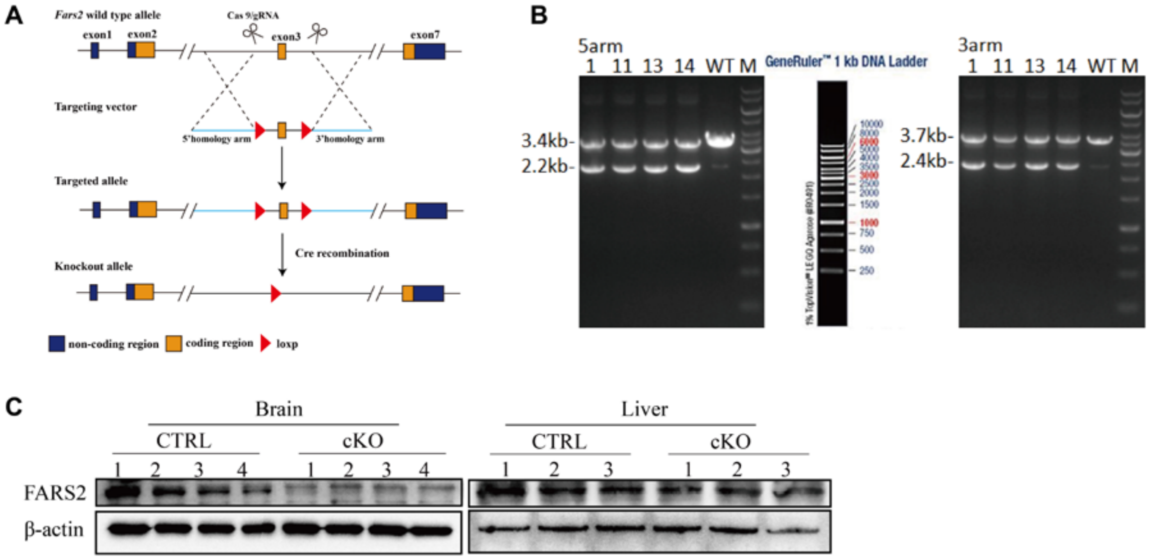


**Figure S3. Establishment of conditional neural-specific *Fars2* knockout-mouse model.**

(A) Diagram of using CRISPR/Cas9 system to create heterozygous conditional mutant and knockout (KO) *Fars2* mice. (B) Identification of F1 mice by PCR using primers from 5' homology arm and 3' homology arm. (C) Confirmation of the *Fars2* knockout efficiency in brain and liver by Western blotting.

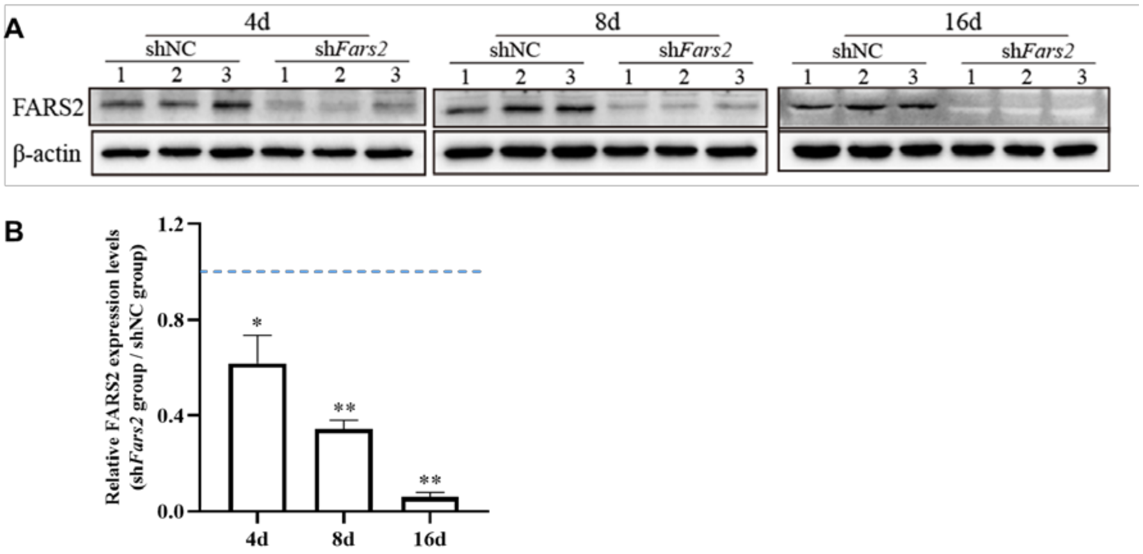


**Figure S4. Establishment of *Fars2*-knockdown neurons *in vitro* using sh*Fars2* lentivirus.**

(A) Confirmation of the *Fars2* knockdown efficiency in primary cultured neurons at 4 d, 8 d and 16 d after infection by Western blotting. (B) Quantification of A.

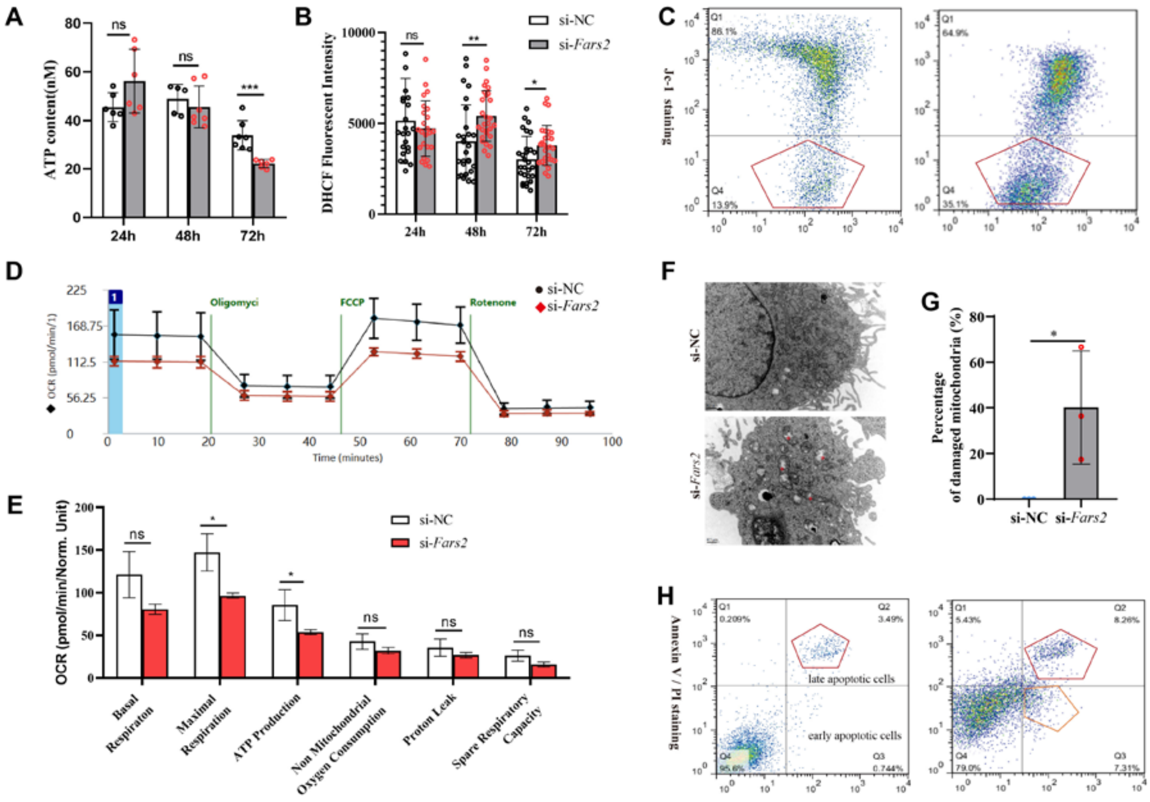


**Figure S5. Mitochondrial dysfunction was confirmed in PC12 cell line.**

(A-B) ATP and ROS levels in control and *Fars2* knocked-down PC12 cells (mean ± SD; two-tailed unpaired t-Test, ns: non-significant; $*P < 0.05$; $**P < 0.01$; $***P < 0.001$; $n$ = 5-7 for ATP; $n$ = 23-28 for ROS). (C) Measurement of mitochondrial membrane potential by flow cytometry using Jc-1 staining revealing increased green fluorescence and decreased red fluorescence. (D) Schematic representation of the oxygen consumption rate for control and *Fars2*-knockdown PC12 cells during MITO stress test using Seahorse XF24 extracellular flux analyzer. (E) Maximal respiration and ATP production were reduced in *Fars2* knocked-down PC12 cells in comparison to control cells (mean ± SD; two-tailed unpaired t-Test, ns: non-significant; $*P < 0.05$; $n = 3$). (F) Transmission electron micrograph of mitochondria in control and *Fars2* knocked-down PC12 cells, mitochondrial cristae with vacuolus are indicated by red asterisks. Scale bar: 0.5 μm. (G) Percentage of mitochondria with abnormal cristae in control and *Fars2* knocked-down PC12 cells (mean ± SD; two-tailed unpaired t-Test, $*P < 0.05$; $n = 3$). (H) Annexin V/ PI staining showed increasd number of early and late apoptosis cells in *Fars2* knocked-down group of cells in comparison to the control.

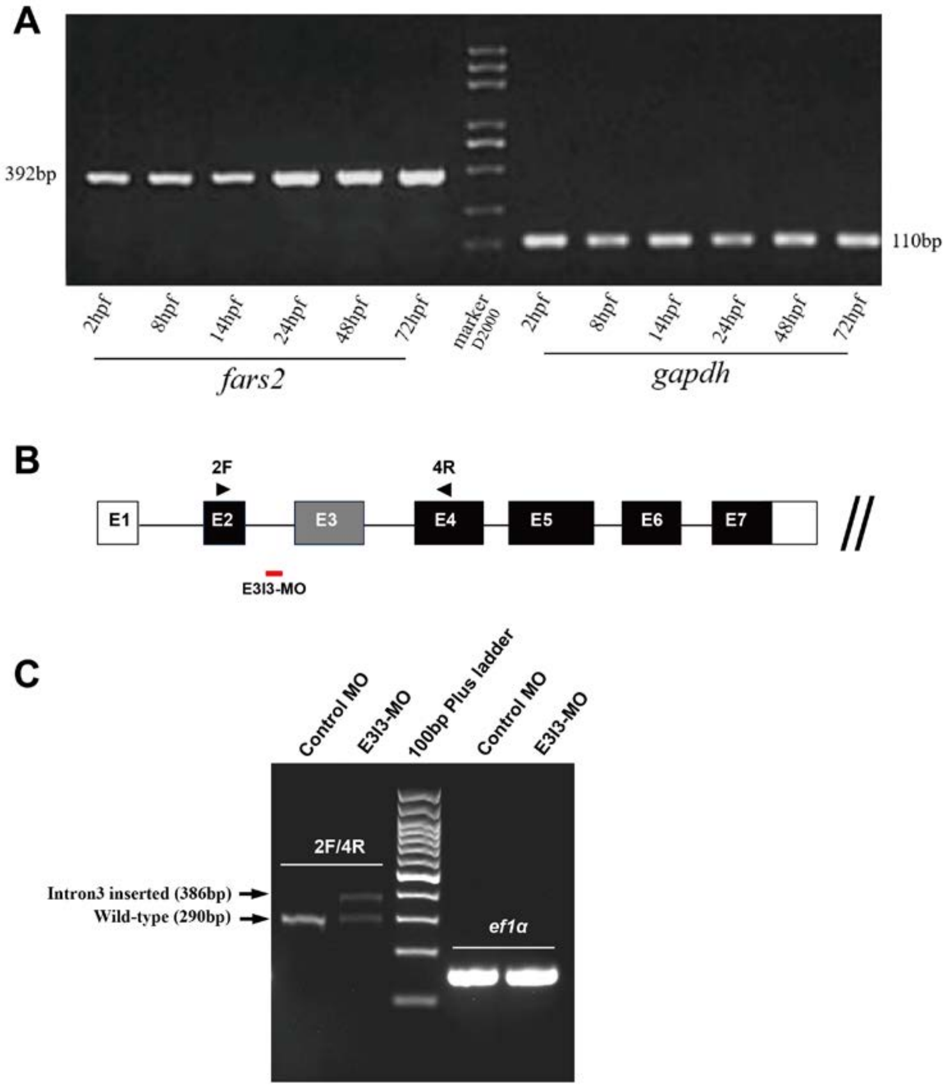


**Figure S6. Effectiveness of *fars2* knockdown was confirmed by RT-PCR.**

(A) Morpholino antisense was designed to block the splice acceptor site of *fars2* exon3 (E3I3-MO) in zebrafish. Primers spanning *fars2* exon 2 and exon 4 were used for RT-PCR analysis for confirmation of the efficacy of the E3I3-MO. (B-C) RT-PCR of *fars2* transcript from Control-MO and E3I3-MO morpholino-injected embryos 2-dpf and 3-dpf, demonstrating insertion of intron 3. dpf, days post fertilization.

**Supplementary Table 1**

| Morpholino Name | Morpholino oligo sequence |
|---|---|
| *fars2*-ATG-MO (ATG-MO) | 5'- CATAGTAGCTGGTCCATAAGCCTCT -3' |
| *fars2*-E3I3-MO (E3I3-MO) | 5'- GAACATGGCAGGATTCCTACCTTCC -3' |
| Standard Control MO | 5'- CCTCTTACCTCAGTTACAATTTATA-3' |

**Supplementary Table 2**

| Primer ID | Sequence |
|---|---|
| *Cytb*-mouse-FP | CCACTCATTCATTGACCTACCT |
| *Cytb*-mouse-RP | GCTCCGTTTGCGTGTATATATC |
| *Nd6*-mouse-FP | GTTAGTGGGTTTGTTGGTTGTT |
| *Nd6*-mouse-RP | CCCAAGTCTCTGGATATTCCTC |
| *Nd3*-mouse-FP | CTACTTCCACTACCATGAGCAA |
| *Nd3*-mouse-RP | TGTTCATTCATATGCTAGGCCT |
| Co3-mouse-FP | ACACTATTAACCCTTGGCCTAC |
| Co3-mouse-RP | AGGCTAGAATGATAGAACGCTC |
| *Atp8*-mouse-FP | CTCATCACAAACATTCCCACTG |
| *Atp8*-mouse-RP | TGGGGTAATGAATGAGGCAAAT |
| *Nd2*-mouse-FP | TTTACCCGCTACTCAACTCTAC |
| *Nd2*-mouse-RP | CATCCTATGTGGGCAATTGATG |
| *Co1*-mouse-FP | ACTCCTACCACCATCATTTCTCC |
| *Co1*-mouse-RP | GGCTAGATTTCCGGCTAGAGG |
| *Co2*-mouse-FP | CTTGGTCTACAAGACGCCAC |
| *Co2*-mouse-RP | CTATTGGCAGAACGACTCGG |
| *Atp6*-mouse-FP | AAGCTCACTTGCCCACTTCC |
| *Atp6*-mouse-RP | GTAAGCCGGACTGCTAATGC |

| | |
|---|---|
| *β-actin*-FP-mouse | GGCTGTATTCCCCTCCATCG |
| *β-actin*-RP-mouse | CCAGTTGGTAACAATGCCATGT |
| *Gli1*-mouse-FP | CCAAGCCAACTTTATGTCAGGG |
| *Gli1*-mouse-RP | AGCCCGCTTCTTTGTTAATTTGA |
| *Ptch1*-mouse-FP | AAAGAACTGCGGCAAGTTTTTG |
| *Ptch1*-mouse-RP | CTTCTCCTATCTTCTGACGGGT |
| *Shh*-mouse-FP | AAAGCTGACCCCTTTAGCCTA |
| *Shh*-mouse-RP | TTCGGAGTTTCTTGTGATCTTCC |
| *Smo*-mouse-FP | GAGCGTAGCTTCCGGGACTA |
| *Smo*-mouse-RP | CTGGGCCGATTCTTGATCTCA |
| *Fars2*-mouse-FP | CTGGTGCTCAGGATCGAATCG |
| *Fars2*-mouse-RP | GCGGATGTCAGGAATGTCGTA |
| *Pax6*-mouse-FP | GTTGGTGTGTTCCCTGTCCT |
| *Pax6*-mouse-RP | ACCGCCCTTGGTTAAAGTCT |
| *Mesp2*-mouse-FP | CGGCGTTCTCTCACCGATG |
| *Mesp2*-mouse-RP | CACCCCACTACTCATGGCTG |
| *Nepn*-mouse-FP | GCTCTCTCTTACCAATGGCCT |
| *Nepn*-mouse-RP | TGATGGGAGTTCCATGAGCCT |
| *Bcl2l13*-mouse-FP | ATGGCGTCCTCTACGACTG |
| *Bcl2l13*-mouse-RP | GGTGAGGGACCTTGTTGTTTC |

| | |
|---|---|
| *Dctn3*-mouse-FP | GCAGGTGGCTTTAGGGAACAT |
| *Dctn3*-mouse-RP | CAGGTATGGCAATGCGATCAA |
| *shha*-zebrafish-FP | CAGATACGAGGGCAAGATAACG |
| *shha*-zebrafish-RP | AGTGGTTCATTACAGAGATGGC |
| *ptch2*-zebrafish-FP | CAGCTTCTACAACTTCATAATGCC |
| *ptch2*-zebrafish-RP | TCCATCCTCTGTGCCATTG |
| *smo*-zebrafish-FP | CTGGCTTTTGGTTTTGTCCTG |
| *smo*-zebrafish-RP | TCTTGATGGCACATTCTGGG |
| *sufu*-zebrafish-FP | GGTCAGAGGCACAGTCATG |
| *sufu*-zebrafish-RP | AGGCACATTGGGATAAGAGTC |
| *gli1*-zebrafish-FP | GCTGATAGAGAAGGAGGAGAGA |
| *gli1*-zebrafish-RP | CTCCACACCGCTGTCATTAT |
| *gli2a*-zebrafish-FP | ACCTCAAGTCCGAAATACCAAG |
| *gli2a*-zebrafish-RP | ACAAGTCTCCTAAATGCTGGG |
| *gli2b*-zebrafish-FP | CACAGACTCACACACCTGAAG |
| *gli2b*-zebrafish-RP | GATCACACAGACCGTCCAATAA |
| *gli3*-zebrafish-FP | GAGAGGTTAAGGCAGGTGAATG |
| *gli3*-zebrafish-RP | AGGCACCCTCACAGAAGTA |
| *ef1α*-zebrafish-FP | GGAAATTCGAGACCAGCAAATAC |
| *ef1α*-zebrafish-RP | GATACCAGCCTCAAACTCACC |

| | |
|---|---|
| *gapdh*-zebrafish-FP | CCAACTGCCTGGCTCCTT |
| *gapdh*-zebrafish-RP | CCCATCAACGGTCTTCTGTG |
| *fars2*-zebrafish-FP | AGAATTATTACCTGAACCGCA |
| *fars2*-zebrafish-RP | CCATCTTATTATCAGCACCA |

**Supplementary Table 3**

| **ShRNA** | **Sequence** |
|---|---|
| shNC(scramble) | GCCTAAGGTTAAGTCGCCCTCGTTCAAGAGACGAGGGCGACTTAACCTTAGGC |
| sh*Fars2* | GGGACAACTATTACTTGAATCTTCAAGAGAGATTCAAGTAATAGTTGTCCC |

**Supplementary Table 4**

| **SiRNA** | sense(5'-3') | antisense(5'-3') |
|---|---|---|
| siCtrl | UUCUCCGAACGUGUCACGUTT | ACGUGACACGUUCGGAGAATT |
| si*Fars2* | CUGUGAAGCUUGUGGAAUUTT | AAUUCCACAAGCUUCACAGTT |

**Supplementary Table 5**

**Proportion of phenylalanine in 5 mitochondrial complexes in human, mouse, zebrafish and rat**

| Species | | Human | Mouse | Zebrafish | Rat |
|---|---|---|---|---|---|
| COMPLEX I | ND1 | 16/318 | 20/318 | 17/324 | 19/318 |
| | ND2 | 14/347 | 14/345 | 10/348 | 11/345 |
| | ND3 | 8/115 | 9/115 | 9/116 | 9/115 |
| | ND4 | 20/459 | 16/460 | 16/460 | 17/459 |
| | ND4L | 3/98 | 5/97 | 5/98 | 6/98 |
| | ND5 | 38/603 | 44/607 | 41/606 | 38/609 |
| | ND6 | 8/174 | 11/172 | 13/172 | 17/172 |
| Total | | 107/2114 (5.061%) | 119/2114 (5.629%) | 111/2124 (5.226%) | 117/2116 (5.529%) |
| COMPLEX III | CYTB | 24/380 | 29/381 | 32/380 | 31/380 |
| Total | | 24/380 (6.316%) | 29/381 (7.612%) | 32/380 (8.421%) | 31/380 (8.158%) |
| COMPLEX IV | CO1 | 41/513 | 43/514 | 42/516 | 42/514 |
| | CO2 | 10/227 | 7/227 | 11/230 | 7/227 |
| | CO3 | 23/261 | 22/261 | 22/261 | 21/261 |
| Total | | 74/1001 (7.393%) | 72/1002 (7.186%) | 75/1007 (7.448%) | 70/1002 (6.986%) |
| COMPLEX V | ATP6 | 9/226 | 14/226 | 14/227 | 13/226 |
| | ATP8 | 1/68 | 1/67 | 4/54 | 4/67 |
| Total | | 10/294 (3.401%) | 15/293 (5.119%) | 18/281 (6.406%) | 17/293 (5.802%) |

**Supplementary Movie 1**

**Locomotor behavioral tests on 54-hpf larvae.** The stereotypic escape response of fars2 morphant was greatly reduced or absents compared to control larvae.
